# GBS-derived SNP catalogue unveiled wide genetic variability and geographical relationships of Italian olive cultivars

**DOI:** 10.1038/s41598-018-34207-y

**Published:** 2018-10-26

**Authors:** Nunzio D’Agostino, Francesca Taranto, Salvatore Camposeo, Giacomo Mangini, Valentina Fanelli, Susanna Gadaleta, Monica Marilena Miazzi, Stefano Pavan, Valentina di Rienzo, Wilma Sabetta, Luca Lombardo, Samanta Zelasco, Enzo Perri, Concetta Lotti, Elena Ciani, Cinzia Montemurro

**Affiliations:** 1 CREA Research Centre for Vegetable and Ornamental Crops, Pontecagnano Faiano, Italy; 2 SINAGRI S.r.l. - Spin Off of the University of Bari “Aldo Moro”, Bari, Italy; 3 Department of Agricultural and Environmental sciences, University of Bari “Aldo Moro”, Bari, Italy; 4 Department of Soil, Plant and Food Sciences, University of Bari “Aldo Moro”, Bari, Italy; 5 Center for Agriculture, Food ad Environment (C3A), University of Trento, San Michele all’Adige, Italy; 6 CREA Research Centre for Olive, Citrus and Tree Fruit, Rende, Italy; 7 Department of the Sciences of Agriculture, Food and Environment, University of Foggia, Foggia, Italy; 8 Department of Biosciences, Biotechnologies and Biopharmaceutics, University of Bari “Aldo Moro”, Bari, Italy

## Abstract

Information on the distribution of genetic variation is essential to preserve olive germplasm from erosion and to recover alleles lost through selective breeding. In addition, knowledge on population structure and genotype–phenotype associations is crucial to support modern olive breeding programs that must respond to new environmental conditions imposed by climate change and novel biotic/abiotic stressors. To further our understanding of genetic variation in the olive, we performed genotype-by-sequencing on a panel of 94 Italian olive cultivars. A reference-based and a reference-independent SNP calling pipeline generated 22,088 and 8,088 high-quality SNPs, respectively. Both datasets were used to model population structure via parametric and non parametric clustering. Although the two pipelines yielded a 3-fold difference in the number of SNPs, both described wide genetic variability among our study panel and allowed individuals to be grouped based on fruit weight and the geographical area of cultivation. Multidimensional scaling analysis on identity-by-state allele-sharing values as well as inference of population mixtures from genome-wide allele frequency data corroborated the clustering pattern we observed. These findings allowed us to formulate hypotheses about geographical relationships of Italian olive cultivars and to confirm known and uncover novel cases of synonymy.

## Introduction

Cultivated olive tree (*Olea europaea L. subsp. europaea var. europaea*) is believed to originate from the wild oleaster (*Olea europaea L. subsp. europaea var. sylvestris*) in the north Levant, a region corresponding to the modern Syrian-Turkish border^1–4^. It is still under debate whether independent domestication events have occurred in the Mediterranean basin or whether it represents a secondary olive diversification centre^5–7^.

Olive was introduced in Southern Italy, first by Phoenicians and, later, by Greek colonization of the region^8,9^. Then, it gained a considerable economic importance with Romans, who disseminated olive cultivation and oil processing facilities all around the Mediterranean basin^10,11^.

Olive cultivars grown today were selected and carried over major migration routes by clonal propagation and grafting. Such migration events were particularly complex and ultimately led to confusion over cultivar nomenclature and identity, resulting in a large number of homonymies, synonymies and errors in the naming of cultivars^12^.

Information on genome-wide patterns of genetic variation and knowledge on population structure of olive germplasm is essential to define priorities for management and conservation of gene pools, to develop new sustainable cropping systems^13,14^ and to study the impact of domestication on olive tree genetic variability.

Investigations into the genetic consequences of domestication and breeding have been already successfully performed on several long-lived perennials. By exploiting a few dozen microsatellite markers, different research groups have assessed the level of genetic variation as consequence of domestication and crop improvement as well as the spatio-temporal origin and the spread of almond^15^, apricot^16^, apple^17^ and olive^18,19^ trees. Using different methods, Myles, *et al*.^20^ applied the Vitis9kSNP array to characterize a *Vitis vinifera* germplasm collection on a genome-wide scale and to infer the domestication and breeding history by evaluating patterns of population structure.

In addition to knowledge on genetic diversity, which is shaped by natural and human-derived processes, genotype-phenotype association is a key prerequisite to modern breeding programs. New challenges for olive breeding are related to (i) the increasing ecological impact of climate change and associated abiotic stresses and (ii) new or resurgent pests and diseases. Among the latter, the emergence of the bacterium *Xylella fastidiosa* has caused severe decline of olive trees in Apulia (Southern Italy)^21–23^.

Morphological characterisation, traditionally used for the assessment of the genetic diversity across olive germplasm collections, has been recently paralleled by the use of molecular markers^24,25^. Different types of DNA markers, namely simple sequence repeats (SSRs)^26–30^, amplified fragment length polymorphism (AFLP)^31–35^ and single nucleotide polymorphisms (SNPs)^36–38^, have been till now used to dissect olive genetic variability.

Compared to other types of DNA markers, SNPs have some advantageous features. They are common and found throughout the genome, stable (i.e. are less mutable), and readily assayed using high-throughput genotyping protocols and automated data analysis. Furthermore, adjacent SNPs in haplotype blocks that tend to be inherited together can be exploited in genetic dissection of complex traits^39^.

Recent progress in next-generation sequencing (NGS) has made SNP discovery cost-effective. Although SNP markers can be observed through various experimental protocols, at present, genotype-by-sequencing (GBS) is the most popular approach for SNP identification in plants^40–42^. In the last few years, GBS has been largely used in species with a reference genome to discover new SNP markers and to develop mapping populations^43–45^, to assess genome-wide diversity and linkage disequilibrium^46–48^, and to perform association mapping studies^49–51^.

Although GBS has been mostly applied to species with complete, near-complete or partial reference genomes, different SNP calling pipelines have been developed to apply GBS to species with limited genomic information^52–54^. Indeed, interesting studies have been successfully carried out in species lacking a reference genome such as switchgrass, oat, blackcurrant, hop, alfalfa and sugarcane^53,55–59^.

To our knowledge, two studies based on GBS and on the reference-independent SNP calling pipeline Stacks^52^ have been performed in olive. These studies aimed at the construction of high-density genetic linkage maps as a resource for locating QTL (Quantitative trait *loci*) associated with agronomically important traits and for genome scaffolding^60,61^.

Herein, we describe the first genome-wide diversity study on a collection of 94 cultivars representative of Italian olive germplasm. Italy has the second-highest level of olive oil production in the World^10,62^ and represents a diversity centre with more than 500 different cultivars grown across its territory^12,63^. This richness in biodiversity was well documented by Hatzopoulos, *et al*.^64^ and by Owen, *et al*.^65^, who described the wide genetic variability for a large number of bio-agronomic traits in Italian olive germplasm. We adopted two different SNP calling procedures: the first one is based on the TASSEL-GBS pipeline and on the partial *O. europaea* genome sequence released by Cruz, *et al*.^66^; the second relies on the reference-independent TASSEL Universal Network Enabled Analysis Kit (UNEAK) pipeline^53^. The extensive catalogue of SNPs we developed was used to (i) measure genetic variation and establish the relationships among all individuals across the population as independently assessed by a parametric (STRUCTURE^67^) and a non-parametric (AWclust^68^) population structure analysis software; (ii) resolve cases of synonymy in olive germplasm and (iii) formulate hypotheses about the geographical relationships and spread of olive cultivars on Italian territory.

## Results

The GBS analysis performed by Illumina sequencing generated ~247 M reads, on average 2.6 M reads per sample. GBS sequence tags were merged into a single master tag file including 1.4 M reads. Two SNP calling pipelines were run, namely TASSEL-UNEAK and TASSEL-GBS.

### Diversity analysis via the TASSEL-UNEAK SNP calling pipeline

**Figure 1:**
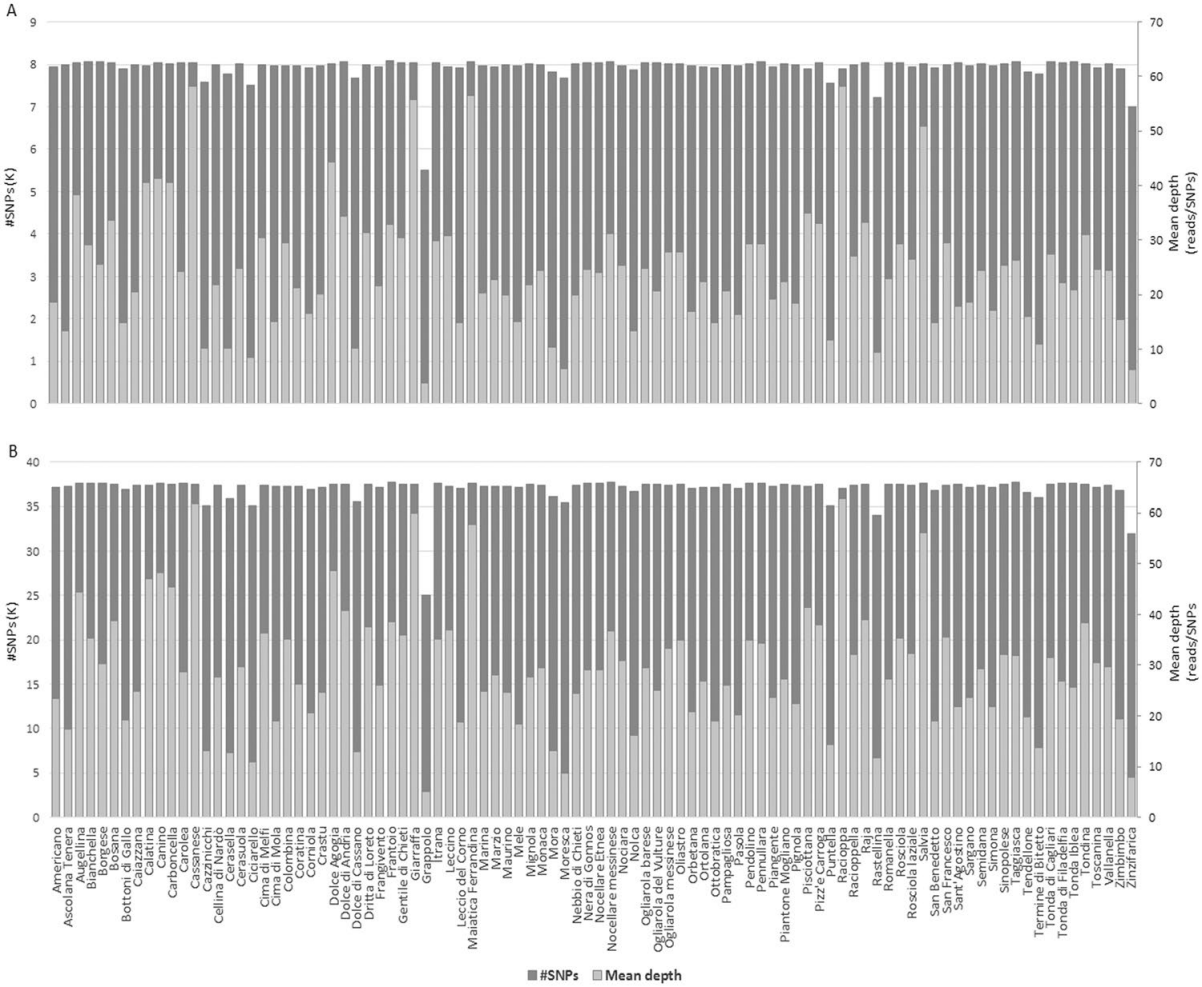
Overlapping bar charts showing SNP count and mean depth per cultivar. (**A**) TASSEL-UNEAK. (**B**) TASSEL-GBS.

The reference-free TASSEL-UNEAK pipeline called 81,820 unfiltered SNPs. By using the filtering criteria described in Methods, the number of SNP *loci* was reduced to 8,088. In Fig. 1A, the mean depth of coverage and the number of SNPs per cultivar are reported. Transitions (Ti) were more abundant (63.1%) than transversions (Tv) (36.9%), with a Ti/Tv ratio of 1.7. The most and the least frequent substitutions were C→T (32.65%) and C→G (5.2%), respectively (see Supplementary Fig. S1). SNP calling revealed that the majority of SNPs were homozygous either for the reference (61%) or the alternate allele (7.5%); on average, 29.7% SNP *loci* were heterozygous, whereas only 1.8% of missing data were observed (see Supplementary Fig. S2A).

**Figure 2:**
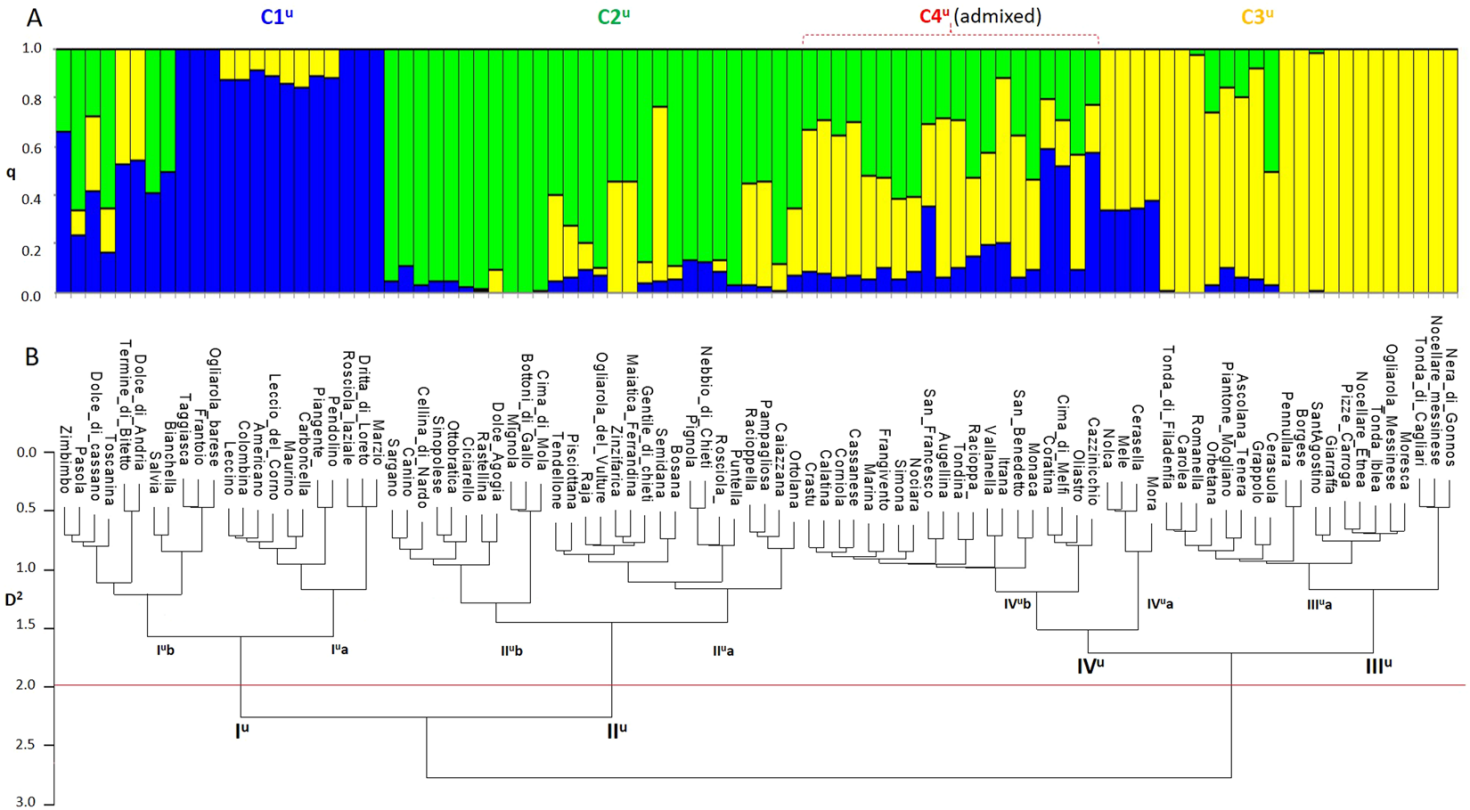
Genetic diversity assessment of 94 *Olea europaeae* cultivars using 8,088 high-quality SNP markers called by TASSEL-UNEAK (^u^). (**A**) Bar-plot describing population structure estimated by STRUCTURE. Population was divided into three clusters plus a cluster of admixed cultivars (C4^u^). Each bar is separated into K coloured segments each representing the ancestry q_i_ proportion in each individual. (**B**) AWclust dendrogram plot showing four main sub-populations. D2 indicates allele sharing distance.

STRUCTURE and Structure Harvester analyses indicated that the germplasm collection genotyped in this study could be divided into three clusters (K = 3; see Supplementary Fig. S3A; Fig. 2). Cluster C1^u^, cluster C2^u^ and cluster C3^u^ include 15, 27 and 29 cultivars respectively; the remaining 23 cultivars are classified as admixed. A clear separation between cluster C1^u^ and cluster C3^u^ can be made on the basis of drupe weight (see Supplementary Table S1). Indeed, cluster C1^u^ includes cultivars with drupe size and weight clearly smaller than those in cluster C3^u^. Pair-wise fixation index (F_ST_) estimate was 0.134 between cluster C1^u^ and C3^u^; 0.093 between cluster C1^u^ and C2^u^ and 0.104 between cluster C2^u^ and C3^u^. The expected heterozygosity was 0.62, 0.84 and 0.72 within cluster C1^u^, C2^u^ and C3^u^, respectively.

Population structure was also investigated using AWclust. Based on Gap Statistics, the most likely number of sub-populations was four (K = 4) (see Supplementary Fig. S4A). As general rule, the hierarchical clustering by AWclust reflects drupe weight variation across cultivars and it is comparable to the Bayesian clustering by STRUCTURE (Fig. 2).

The dendrogram by AWclust displays two primary nodes and four clusters (Fig. 2). Cluster I^u^ groups 22 olive varieties with an average drupe weight  = 2.43 g ± 0.81. This cluster can be split into two sub-clusters, each including varieties from a specific geographical area of cultivation: I^u^a (cultivars from Central Italy) and I^u^b (cultivars from Apulia).

Cluster II^u^ includes 28 cultivars and it is separated into two clades: II^u^a and II^u^b. Cultivars in clade II^u^a have drupes of medium weight (average of 2.84 g ± 0.86) and fall into distinct branches corresponding to different Italian regions. Clade II^u^b groups cultivars with small drupes (=1.65 g ± 0.28) typically cultivated in Calabria and in “Salento”, an area located in the South of Apulia region.

Cluster III^u^ comprises 20 cultivars with the highest drupe weight (average  = 5.28 g ± 1.62), mainly cultivated in the two insular Italian regions. Finally, cluster IV^u^ includes two clades for a total of 24 cultivars with drupes of medium weight (average of 3.44 g ± 0.99): IV^u^a groups four varieties cultivated in Apulia (Mora, Cerasella, Mele, Nolca), while IV^u^b includes cultivars mainly cultivated in Sicily and Calabria.

The one-way analysis of variance (ANOVA), that was used to determine whether there are any statistically significant differences between the means of drupe weight of cultivars in AWclust and STRUCTURE groups, confirms significant differences among the means (see Supplementary Table S2).

### Diversity analysis via the TASSEL-GBS SNP calling pipeline

The master tags were aligned to the olive reference genome^66^. Approximately 54% of the reads mapped uniquely to the reference, while 15.9% aligned to multiple positions and 30.6% of GBS sequence tags failed to align. In total, the reference-based TASSEL-GBS pipeline yielded 225,919 unfiltered SNPs. Of these, 37,792 were retained for downstream analyses after applying the filtering criteria described in the methods section. Figure 1B reports the mean depth of coverage and the number of SNPs per cultivar. Taking advantage of the genomic coordinates of olive gene models, 10,087 (26.7%) and 27,705 (73.3%) SNPs were located in genic and intergenic regions, respectively. More precisely, 2,690 SNPs (26.75%) fell within annotated exons, affecting a total of 1,302 genes.

The majority of the identified SNPs (64.6%) were transitions (Ti), with a Ti/Tv ratio of 1.82. The most and the least frequent substitutions were C→T (32.7%) and C→G (5.2%), respectively (see Supplementary Fig. S1).

We also applied LD pruning to the 37,792 high-quality SNPs in order to resolve population genetic structure. This resulted in 22,088 SNPs, of which, the vast majority was homozygous for the reference (74.8%), whereas a few *loci* were scored for the alternate allele (5.9%); ~16.8% of SNPs were heterozygous and only 2.2% were the missing data (see Supplementary Fig.S2B).

Ten SNP *loci* were randomly selected in three different cultivars and were validated by PCR amplifications and Sanger sequencing. All the polymorphisms identified *in silico* were confirmed (see Supplementary Table S3).

The dataset of 22,088 SNPs, called by using cv. Farga as reference genome^66^, was used to categorize cultivars into clusters based on their genetic structure.

**Figure 3:**
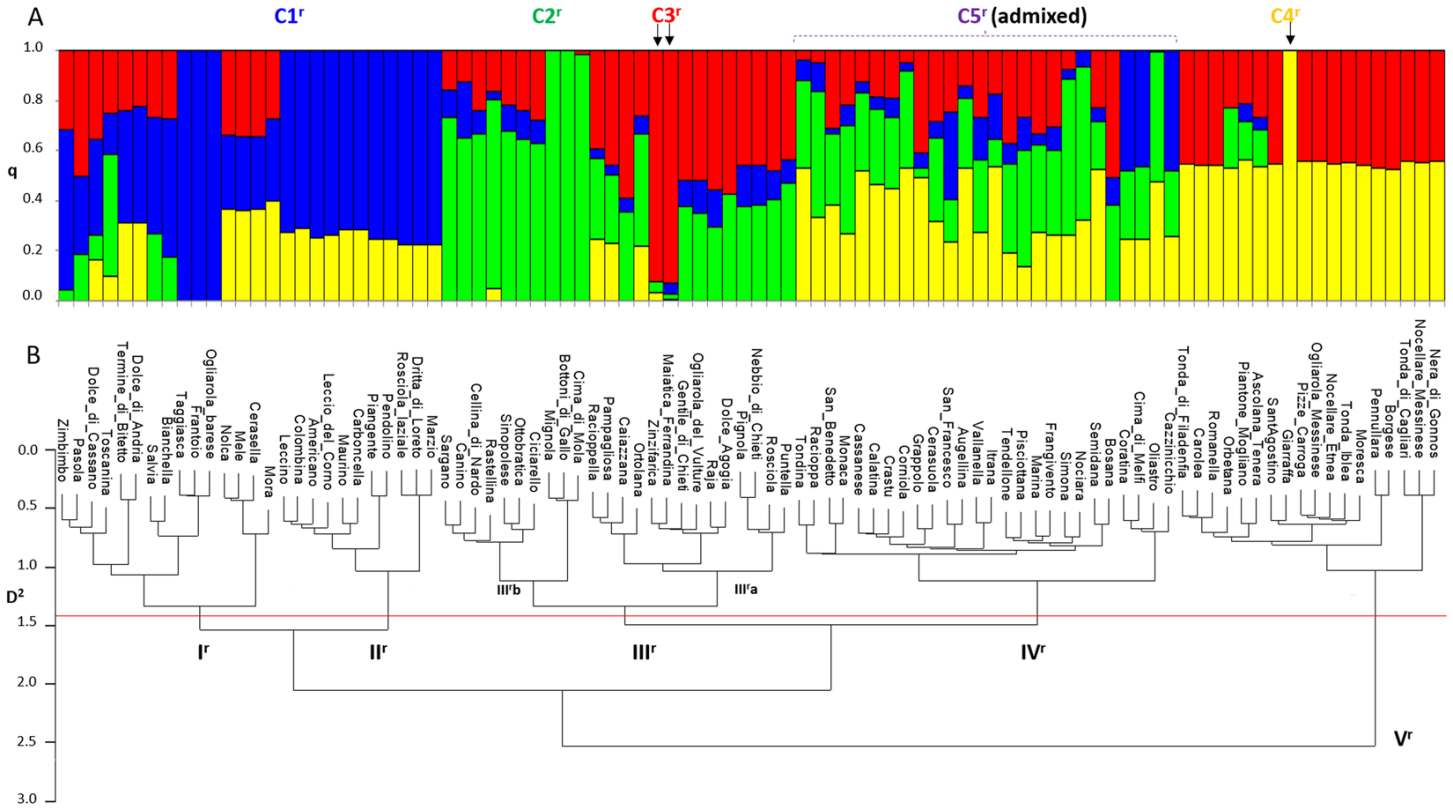
Genetic diversity assessment of 94 *Olea europaeae* cultivars using 22,088 high-quality SNP markers called by TASSEL-GBS (^r^). (**A**) Bar-plot describing population structure estimated by STRUCTURE. Population was divided into four clusters plus a cluster of admixed cultivars (**C**5^r^). Each bar is separated into K coloured segments each representing the ancestry q_i_ proportion in each individual. Black arrows indicate bars corresponding to cultivars included in clusters C2 and C3. (**B**) AWclust dendrogram plot showing five main sub-populations. D2 indicates allele-sharing distance.

Structure Harvester indicated K = 6 as the optimal number of sub-populations for the germplasm collection, immediately followed by K = 4 (see Supplementary Fig. S3B Fig. 3). Considering that, at both K = 6 and K = 4, most of the cultivars fell in the admixed group at q_i_ ≥ 0.60 (68 and 64 varieties, respectively), we decided to divide the population under investigation into four sub-populations since this best fit with AWclust clustering. At q_i_ ≥ 0.60, Giarraffa was the only accession included in the cluster C4^r^. Clusters C1^r^, C2^r^ and C3^r^ include 15, 12 and 2 varieties, respectively. Pair-wise fixation index (F_ST_) estimated values were: 0.130 between cluster C2^r^ and C1^r^, 0.184 between cluster C1^r^ and C3^r^ and 0.100 between cluster C2^r^ and C3^r^.

The optimal number of sub-populations detected by AWclust was K = 5 (see Supplementary Fig. S4B). The grouping and distribution of olive cultivars into five clusters overlapped to a larger extent with those obtained by TASSEL-UNEAK, as previously described. Cultivars were clustered into two main clades including 76 and 18 varieties, respectively (Fig. 3). The clade with the largest number of individuals is split into four clusters, all including cultivars with average drupe weight ≤3.50 g. Clusters I^r^ and II^r^ collects 15 and 11 cultivars with a wide range of drupe weight (average of 3.07 g ± 1.14 and of 2.14 ± 0.33) cultivated in Apulia and Central Italy, respectively. Clusters III^r^ (2.19 ± 0.81) and IV^r^ (3.27 ± 0.90), comprising 24 and 26 cultivars, correspond to clusters II^u^ and IV^u^, respectively. Cluster V^r^ is clearly separated from the rest (Fig. 3). This cluster groups 18 cultivars with the highest drupe weight (an average of 5.43 g ± 1.45) mainly cultivated in Sicily and Sardinia and corresponds to cluster III^u^ previously described (Fig. 2).

The one-way ANOVA resulted in statistically significant differences between the means of drupe weight of cultivars in AWclust and STRUCTURE groups (see Supplementary Table S2).

### Degree of allele sharing by *identity-by-state* and inference of population mixtures

**Figure 4:**
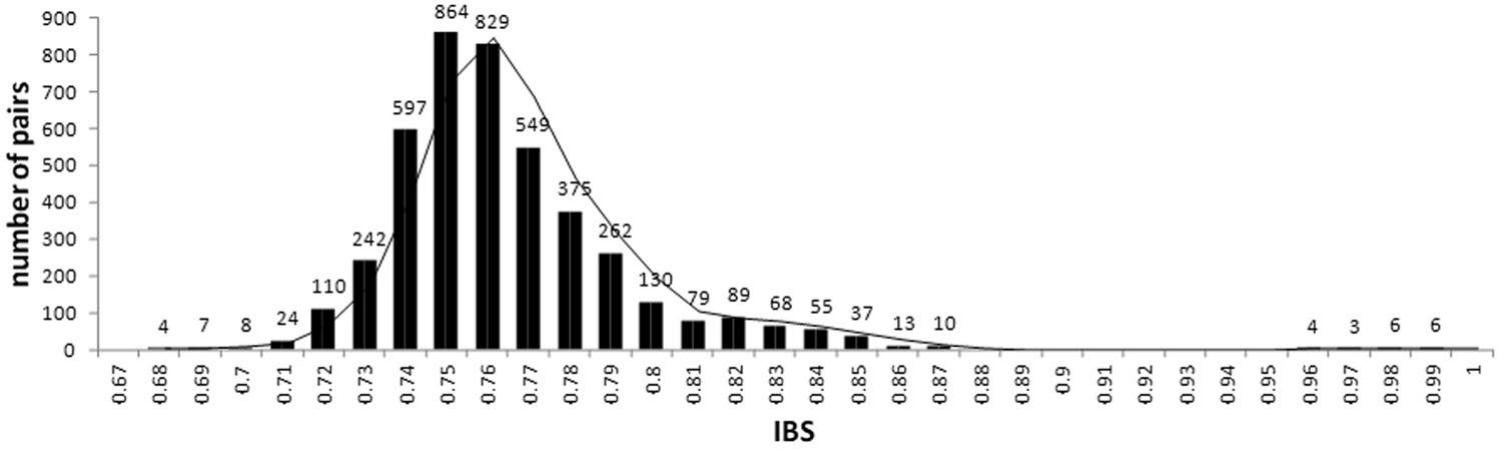
Distribution of identity-by-state (IBS) allele sharing values amongst 94 olive tree cultivars determined by the analysis of 22,088 unlinked single nucleotides polymorphisms.

Relationships among the 94 *Olea europaea* cultivars were also explored by estimating identity-by-state (IBS) allele-sharing values for all pair-wise comparisons using 22,088 unlinked SNPs. The frequency distribution of IBS estimates in Fig. 4 shows that most of the cultivars falls in the bin from 0.74 to 0.77 and that only 19 pairs of cultivars have allele-sharing values >0.95 (see Supplementary Table S4).

A multidimensional scaling (MDS) plot of genome-wide IBS pair-wise distances (see Supplementary Fig. S5) shows a clear separation of the cultivar into 3 groups, while members of two other groups are scattered in the multidimensional space. The MDS proximity matrix confirms to some extent the clustering pattern observed with STRUCTURE and AWclust, respectively.

The tree-based approach implemented in TREEMIX^69^ was chosen in order to infer patterns of population mixtures from genome-wide allele frequency data and to test the presence of gene flow (i.e. the transfer of genetic variation from one sub-population to another). TREEMIX was run on the dataset described above, with olive cultivars grouped into 4 arbitrary sub-populations (i.e. the clusters (C1^u^, C2^u^, C3^u^, C4^u^) identified following population structure definition based on TASSEL-UNEAK SNP markers).

Analysis of the TREEMIX log-likelihood values for 0 to 3 migrations revealed that the most predictive model (i.e. that had the highest log-likelihood) assumed the presence of 2 migration events (see Supplementary Table S5). A strong signal of gene flow and/or shared ancestry was inferred between C1^u^ and C4^u^ (0.49) and C3^u^ and C4^u^ (0.28).

This indicates an exchange of genetic material between sub-populations C1^u^ and C4^u^ as well as C3^u^ and C4^u^. What was observed was expected since C4 includes only admixed genotypes. In contrast, we observed negligible gene-flow between C1^u^, C2^u^, C3^u^.

### Linkage disequilibrium

**Figure 5:**
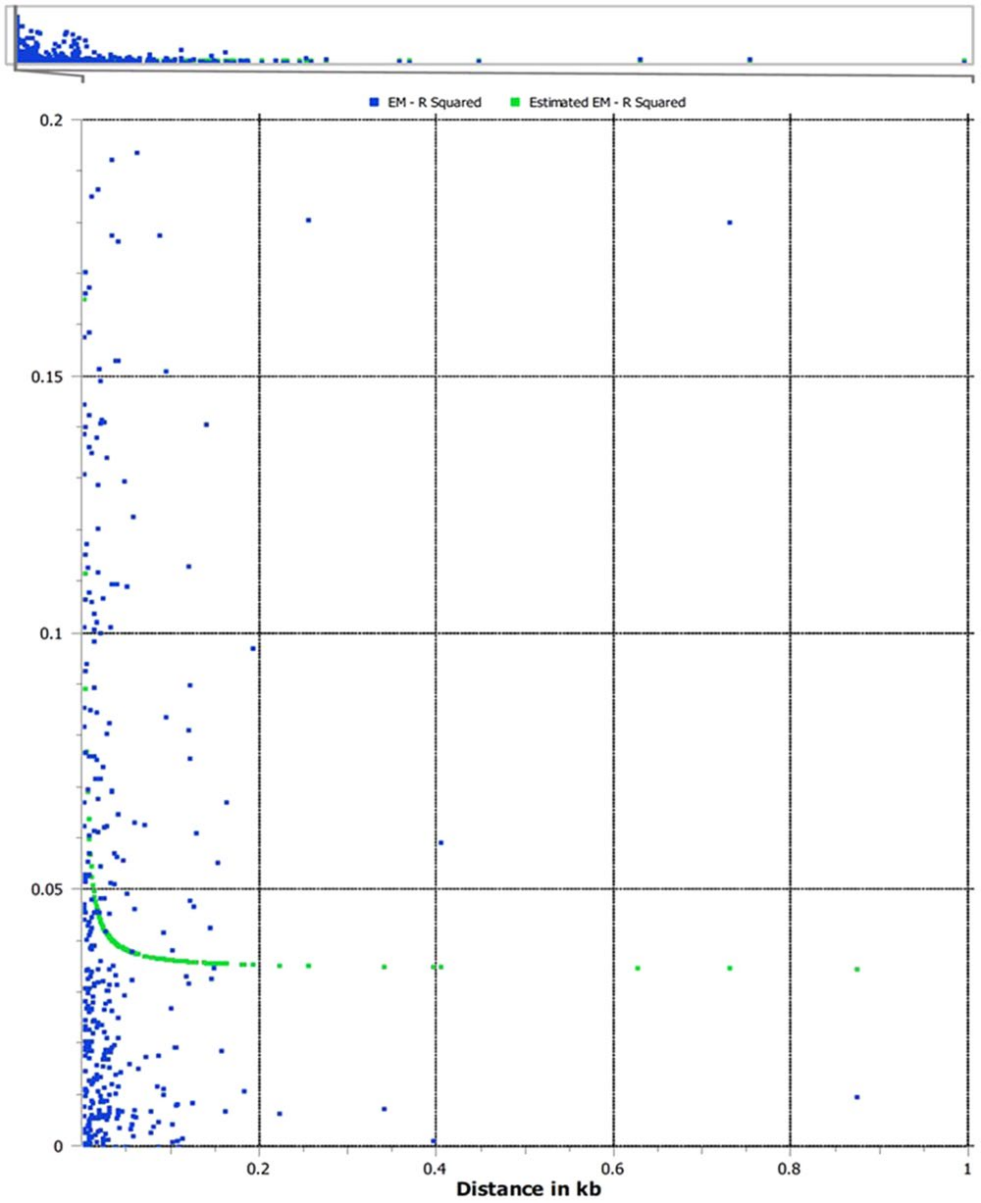
Scatter plot showing linkage disequilibrium decay (r^2^) calculated using a subset of the 22,088 SNPs called by TASSEL-GBS located in the 30 longest olive scaffolds.

Linkage disequilibrium was calculated for all possible combination of pairs (r^2^) of 22,088 SNPs detected by TASSEL-GBS. Taking into account that these SNPs are located on more than 5,000 scaffolds that differ in size, LD decay was estimated considering only those SNP markers identified in the 30 longest scaffolds (see Supplementary Table S5). LD estimation suggested a very rapid decay, with average r^2^ dropping to 0.05 within 0.025 kb (Fig. 5).

## Discussion

A key requirement for progress in any modern olive tree breeding program is to capture the widest possible genetic variability across germplasm collections as well as to investigate genotype–phenotype associations for the basic understanding of adaptive traits.

To this end, SNP markers are a valuable resource to enhance our knowledge on the genetic structure of *O. europaea* populations and to carefully dissect genetic variability within germplasm collections. The latter is a necessary step for the conservation and future utilization of olive gene pools and for the recovery of alleles left behind by selective breeding. Such reservoir of alleles provides a powerful tool for breeders to undertake efficient breeding programs for the development of novel varieties best suited to new cropping systems and biotic and abiotic stresses^70^. To the best of our knowledge, no studies have been performed yet on Italian olive germplasm based on high-throughput SNP discovery.

Within this motivating context, we performed a genome-wide diversity study on a panel of 94 olive cultivars representative of Italian germplasm via genotype-by-sequencing. We believe that the use of different analytical approaches to detect SNP variation and estimate population structure and genetic relationships makes our work relevant and valuable from a methodological point of view.

By using a reference-based and a reference-independent SNP calling pipeline we developed an extensive catalogue of SNPs used to model population structure via parametric and non parametric-based clustering and investigate relationships among Italian olive cultivars. Furthermore, our results unveil cases of possible synonymies (see Supplementary Tables S4,S7) and support new hypotheses on the geographical relationships among olive varieties cultivated in Italy.

It is well known that the availability of a reference genome can facilitate GBS data analysis, although several reference*-*independent SNP calling pipelines have been successfully applied for genetic diversity studies. We used two different SNP calling pipelines, namely, TASSEL-UNEAK (reference-independent) and TASSEL-GBS (reference-based). Even if the current olive tree genome assembly is far from being chromosome-scaled (it is composed of more than 5,000 scaffolds covering 1.31 Gb out of an estimated genome size of 1.38 Gb)^66^ and despite the lack of a “gold standard” structural and functional annotation, we still used this reference genome for the *de novo* discovery of SNP markers via GBS. As expected, TASSEL-GBS outperformed TASSEL-UNEAK with respect to the number of high quality SNPs (22,088 vs. 8,088). The 3-fold difference in the number of SNPs could be influenced by the more stringent parameters used in absence of a reference genome^53^. Conversely, the mean depth of coverage, and the frequency of reference and effect (or alternative) SNP alleles per cultivar were comparable between the pipelines (Figs 1 and S1). This result further endorses that GBS is a valid and robust tool for SNP discovery even when a reference genome is lacking and that reference-independent SNP calling pipelines can be definitely valuable in underutilized, neglected, or orphan crops.

As mentioned before, a high number of master tags were generated, however only 53.6% of them aligned to the reference genome following the GBS tag-to-reference genome alignment step in TASSEL-GBS. This is not surprising. Indeed, missed alignments (false negative) can be ascribed to (i) distance between DNA sequences that might have prevented read-to-genome-alignments especially when very stringent alignment parameters were used to minimize the number of multiple alignments; (ii) the incomplete nature of the reference genome; (iii) regions with lower quality sequences; (iv) presence of reads from organelle genomes.

In this study we applied two complementary clustering methods (a parametric Bayesian clustering, that assume Hardy-Weinberg equilibrium and linkage equilibrium among *loci* in individuals of the sample population, and a non-parametric distance-based hierarchical clustering, that it is based on a matrix of pair-wise allele sharing distances between all of the individuals in the dataset) to assess genetic diversity and establish relationships among individuals in the population under investigation. As previously discussed elsewhere, the two methods were found to corroborate each other remarkably well^48^.

The comparison between the two AWclust*-*derived dendrograms (Figs 2B,3B) shows that SNPs called both by TASSEL-GBS and TASSEL-UNEAK usually assign cultivars to similar clusters with three minor differences.

The first one affects olive cultivars with the highest drupe weight. They were assigned to distinct clusters with low overlap among their elements. However, when we examine the dendrogram developed on the basis of the SNPs identified by TASSEL-GBS, a significant clustering adjustment is observed. All olive cultivars with the highest drupe weight (cluster V^r^) originate from a single ancestor node, which clearly separate them from the remaining cultivars with medium and low drupe weight. This finding suggests that the most important parameters which influenced clustering analysis are size and weight of the drupe. This assertion is consistent with results from previous studies, which indicates those as high heritability traits^8,71,72^.

This hypothesis is also supported by the second difference we observed in the clustering. This concerns the clusters to which Cerasuola and Grappolo, characterised by having a medium drupe weight, belong. Based on SNPs called by TASSEL-UNEAK, they unexpectedly grouped in cluster III^u^a with most cultivars characterised by highest drupe weight. Contrariwise, the clustering that relies on SNPs called by TASSEL-GBS assigns these two cultivars in a cluster (IV^r^b) including twenty-two varieties with comparable drupe weight (4–6 g.).

Finally, the third difference we found affects the sub-set of natural sweet Apulian cultivars (Mora, Cerasella, Mele and Nolca), whose fruits are natural debittering on the tree during ripening^73,74^. In the dendrogram in Fig. 2, this sub-set is part of the cluster (IV^u^) that includes cultivars with medium drupe weight. Interestingly, the same sub-set is located into the cluster I^r^ (Fig. 3), where all Apulian “sweet” olive cultivars are placed. This example clearly shows that the AWclust*-*derived dendrogram generated by TASSEL-GBS SNPs does not only group cultivars based on drupe morphological features, but also defines clusters based on the geographical area of cultivation. Indeed, the hierarchical clustering based on SNPs called by TASSEL-GBS resulted more robust and informative compared to the one based on TASSEL-UNEAK.

At first glance, STRUCTURE clustering may look less self-explaining than that made by AWclust. Going further in detail, population structure inferred by TASSEL-UNEAK SNP markers seems to be more profitable compared to that assessed by TASSEL-GBS, which includes a larger number of cultivars with a mosaic of allele frequencies (i.e. admixed ancestry).

The fact that SNPs called by TASSEL-GBS and by TASSEL-UNEAK return several admixed genotypes reveals that Italian olive germplasm has accumulated high level of variability over the centuries. This is supported by the fact that Italy is located in the middle of the Mediterranean basin that is considered a hybrid area between the Eastern and Western zones^3,75,76^ where diversification of cultivated olive tree mainly took place. Furthermore, we cannot ignore that the very high genetic variability in olive tree is especially due to its mating system. Olive tree is an allogamous wind-pollinated species to which self-incompatible cultivars belong^77^. This results in an increase of spontaneous crosses that give rise to olive genotypes with a spectrum of allele frequencies derived from ancestors^78^. Several studies have documented genetic admixture on a local or large scale in olive tree given the out-crossing nature of *O. europaea*^18^. Indeed, gene flow between wild and domesticate forms has been hypothesized to have shaped olive genetic diversity across the Mediterranean basin^9,18,28^.

Many studies based on a small number of SSR and AFLP markers have been carried out to identify synonyms and/or homonyms among Italian olive cultivars, although they do not unequivocally clarify the existing genetic relationships^9,12,79^.

Pair-wise clustering based on IBS as well as allele frequency estimates suggests the occurrence of several cases of synonymy. Many of them have been already described in previous studies based on morphological traits and molecular markers^12,32^, while others were uncovered here for the first time (see Supplementary Tables S4,S7). By fixing IBS values ≥0.95, we found 19 pairs of cultivars that look similar to each other (see Supplementary Table S4). Based on coefficient membership (q_i_; i.e. probability of an individual belonging fully to one ancestral population) it is possible to distinguish several cases of synonymy among the individuals that draws most of their genetic ancestry from different populations. For all these cases, varieties are cultivated in confined geographical areas and it is possible that original names were altered in accordance with local dialects. Interestingly, by fixing q_i_ ≥ 0.97 we observed the following two cases of synonymies: (i) Cima di Mola, Bottoni di Gallo and Mignola (q_i_ = 0.98); (ii) Ogliarola barese, Taggiasca and Frantoio (q_i_ = 1). In both cases, synonymies are certainly not attributable to varieties cultivated in narrow geographical areas. It is well known that genetic improvement of olive is also characterised by vegetative propagation of the most valuable individuals^80^.

With this in mind, we can speculate that some individuals were vegetative propagated by cuttings or clonal propagation and were disseminated by human migrations across the Italian peninsula disregarding the cultivar name. It must however be stressed that cultivars genetically indistinguishable from others could be phenotypically different. This is not surprising since variations in light, altitude, soil composition and water availability could completely change the physiological and morphological aspect of the olive plant^81–83^.

The results we obtained by using SNPs called by TASSEL-GBS and TASSEL-UNEAK and applying two complementary methods for the estimation of genetic diversity indicated a clear and consistent subdivision of the cultivars under investigation into three main groups.

This finding, together with data on patterns of population splits and mixtures, allowed us to formulate hypotheses about the geographical relationships, dissemination and diversification of olive cultivars in Italy. Given the above, we distinguished a sub-population that includes cultivars from Apulia (I^r^ or I^u^a) and Central Italy (II^r^ or I^u^b) that may have evolved from a common ancestor population.

F_ST_ values revealed moderate isolation between cultivars in C1^u^ cluster and all the others. In addition, a strong signal of gene flow between C1^u^ and C4^u^ (that includes admixed genotypes) was observed. The MDS plot of genome-wide IBS pair-wise distances shows that members of the cluster Ir and IVr (i.e admixed genotypes) are scattered in the multidimensional space. On the basis of these further evidences we can state that C1^u^ represents a relatively closed gene pool that exchanged genetic material through inter-breeding with other varieties cultivated in Italy.

Most likely, the C1^u^ population originated from local oleasters intermixed with feral forms and has spread to different Italian regions over time.This hypothesis is supported by Baldoni, *et al*.^9^, who investigated the genetic relationships among wild types and cultivars collected from three Italian regions: Umbria, Sicily and Sardinia.

The authors concluded that Umbrian cultivars have mainly originated by selection from local oleasters. Interestingly, these cultivars are widely disseminated in the regions of north, central and southern Italy and this suggest their ancient origin. Albertini, *et al*.^84^ reported that the modern Italian olive cultivars, localized in Central Italy, could derive from the hybridization between cv. Leccino and Dritta di Loreto with ancestral genotypes. Finally, Muzzalupo, *et al*.^12^ pointed out a high level of gene flow from the varieties cultivated in Central Italy and the others spread throughout the Italian territory.

The second sub-population (Cluster V^r^ or III^u^) includes cultivars mainly from Sicily and Sardinia. We proved that these cultivars never exchanged genetic material with the remaining varieties under investigation. The close relationship between the pools of the two islands suggests that they did not originated from local oleasters but most likely have been introduced into these regions from the outside^9^. This hypothesis is supported by Las Casas, *et al*.^79^, who assessed the genetic diversity of olive cultivars from Sicily (several of which were also analyzed in this study) and other countries of Mediterranean basin. The authors highlighted that Sicilian cultivars clearly separate from other Italian varieties but were grouped with cultivars from Spain and Marocco. Indeed, historical and cultural relationships between Catalan and Sardinian and Sicilian cultures are well known^85^ as well as trading contacts between Italian insular regions and Phoenicians.

The third sub-population (cluster III^r^ or II^u^) could derive from a common ancestor from Magno-Greek origin, since all the cultivars in this sub-population are cultivated in Southern Italy, that was colonized by the Greeks in the eighth century BC^86^. In particular, two sub-clusters can be identified: cluster III^r^a or II^u^a and III^r^b or II^u^b refer to the area of Ionic and Doric influence, respectively. Within the Doric group are cultivars from Salento (Cellina di Nardò) and Calabria (Sinopolese e Ottobratica). All are characterised by small drupes and monumental trees and are subjected to the same cropping system^87^.

The Ionic group includes cultivars originating from the Magno-Greek Ionian cities such as Ferrandina and Metaponto^88^, and, indeed, the most representative cultivar within this group is “Maiatica di Ferrandina”.

It is noteworthy that varieties cultivated in geographically distant but culturally close areas are part of this group. This is probably due to close trading ties between the Etruscans/Italiote populations (Campania: Rosciola, Puntella, Caiazzana; Umbria/Lazio: Dolce Agogia) and the Magno-Greek colonies^89^.

**Figure 6:**
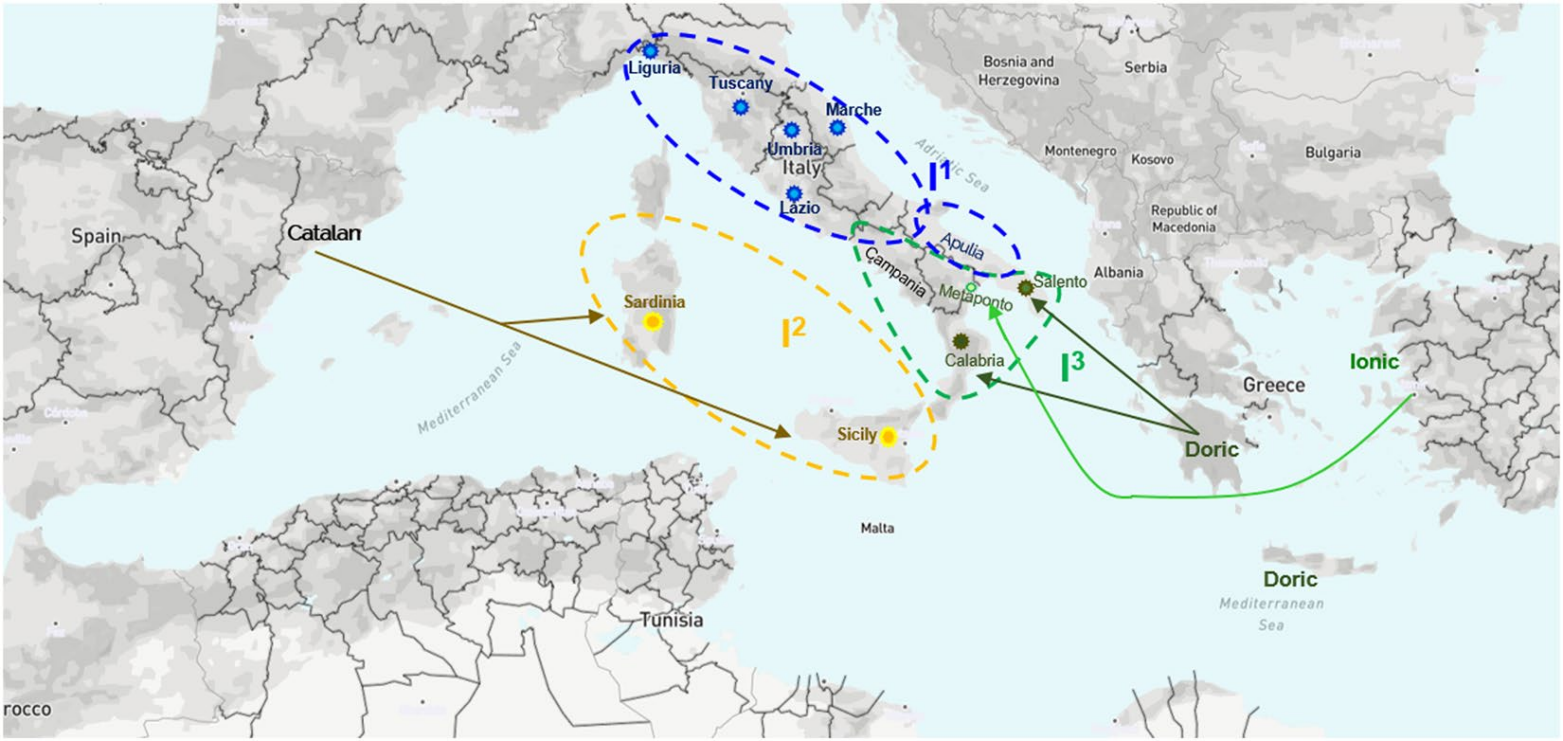
Geographical distribution on Italian territory of three main gene pools we identified via GBS-derived SNP markers in the olive germplasm collection under study. The blue circles (I_1_) encloses all the Italiote cultivars with admixed ancestry. Inside the yellow circle (I_2_) all the cultivars with Catalan origin are placed. Finally, inside the green circle (I_3_) are most of the cultivars of Magno-Greek origin split into varieties from Ionic (dark green stars) and Doric (light green stars) area of influence.

To sum up, we identified three main gene pools, which we named I^1^, I^2^, I^3^. I^1^ represents most of the Italiote cultivars with admixed ancestry; I^2^ consists of cultivars of Catalan origin and I^3^ includes most of the cultivars of Magno-Greek origin (Fig. 6).

Such a grouping reflects to some extent what already observed by Diez, *et al*.^18^ and by Besnard, *et al*.^3^.

According to Besnard *et al*.^3^, the centre of olive origin would be in the North Levant, from which two parallel diversification processes took place, one in the Western and the other in the Eastern part of the Mediterranean basin. In order to verify this hypothesis, Besnard *et al*.^3^ used chloroplast DNA markers to genotype a large collection of cultivars from all over the Mediterranean basin. The authors identified three lines of ancestry tagged as E1, E2 and E3. Line E1 included cultivars from the Eastern Mediterranean (North Levant and Greece), while lines E2/E3 consist of varieties from the Western and Central Mediterranean. A further study based on SSR markers highlighted the existence of three genetic groups of olive cultivars in the Mediterranean basin (tagged as Q1, Q2 and Q3)^18^. Q3 includes cultivars subjected to the first event of domestication occurred in North Levant (which corresponds to line E1), followed by a secondary independent event of domestication in central Mediterranean basin (Q2). Notably, Q2 (which corresponds to lines E2/E3) is a product of admixture between the set of Eastern domesticates (Q3) and Western oleasters. A close genetic relationship between cultivars in Southern Spain (Q1) and the feral forms from the Eastern was observed.

The scenario just outlined for the population under investigation, although supported by different methods of analysis and by literature on the subject, may serve as working hypothesis for subsequent studies.

Herein, we evaluated the extent of LD decay and found that the rapid LD decay inferred by this study is consistent with previous estimates in olive^38^ as well as in other fruit crops^90^. The low extension of LD may be probably due to the self-incompatibility of several olive cultivars: the higher the level of heterozygosity is, then the lower the LD that is counterbalanced by the increasing number of recombination events.

Although our work overlaps to some extent previous studies based on a limited number of AFLP, SSR and SNP markers, we provided much more precise indications on genetic similarity among the cultivars in the germplasm collection thanks to a large genome-wide SNP panel. Indeed, we were able to capture genetic variability at an unprecedented level of detail. This, in turn, allowed pairs of cultivars that look very similar to each other (cases of synonymy) to be identified based on identity-by-state (IBS) computation.

In total agreement with previous studies, we corroborated the evidence that the geographical area of cultivation is a driving force for genetic clustering. The novelty that emerges when allele-frequency distribution histograms by STRUCTURE and dendrograms by AWclust are taken into account is that olive drupe weight plays a major role in structuring genetic diversity in olive.

Finally, we believe that the genome-wide SNP panel we generated and released to the public will be valuable for future genome-wide association studies.

## Methods

### Plant material and DNA extraction

A panel of 94 *Olea europaea* L. var. *sativa* olive cultivars (see Supplementary Table S1) was selected from a large collection of ~500 cultivars corresponding to 85% of the total Italian olive germplasm^12^ grown at the experimental field of CREA Research Centre for Olive, Citrus and Tree Fruit on the Ionian Sea cost of Northern Calabria, Italy (39°37′00′′ North latitude, 16°45′53′′ East longitude, 6 m a.s.l.). Olive trees were spaced with a regular planting pattern of 4 × 6 m. Drupe weight (g) in Supplementary Table S1 was measured considering the average weight of 100 drupes per cultivar.

Such germplasm is considered diverse on a regional scale since each region has gradually selected varieties adapted to environmental, agronomic, cultural and traditional features of the site. With this in mind, the 94 cultivars were selected so that they could represent the whole genetic diversity and phenotypic variability of the original collection. Genomic DNA was extracted from young leaves using the protocol described by Doyle^91^ with minor modifications as follow: after re-suspension in TE 0.1X, 2 volumes of CIA 24:1 were added to the mixture, then 2.5 volumes of 100% ethanol and 1/10 volume of sodium acetate 3 M pH 5.2 were added to force DNA precipitation. DNA quality and concentration were checked by agarose gel 0.8% and Qubit 3.0 fluorometer (Life Technologies, USA).

### Genotyping-by-sequencing and SNP calling

Genotyping-by-sequencing was performed as described by Taranto, *et al*.^48^ using the EcoT22I restriction enzyme. Two different pipelines were run for SNP calling: the reference-independent TASSEL-UNEAK pipeline^53^ and the reference-based TASSEL-GBS pipeline^92^. In the TASSEL-GBS pipeline, master tags (i.e. collapsed sequence tags from each sequence file) were aligned along the olive tree reference genome available at http://denovo.cnag.cat/genomes/olive/download/Oe6/Oe6.scaffolds.fa.gz^66^ using the Burrows-Wheeler Aligner tool (version 0.7.8-r455) with default settings.

Both pipelines produced a VCF file that was subjected to a filtering procedure using VCFtools [version 0.1.13^93^;] with the following parameters: minimum allele frequency (MAF) ≥0.05, max-missing = 0.90, Hardy-Weinberg Equilibrium (hwe) = p ≤ 0.001 and min-mean depth = 5. Single nucleotide InDels were removed. VCFtools were also used to generate various statistics on the dataset under investigation and to add gene annotations to VCF files.

Ten SNP *loci* were selected in three cultivars (i.e. Ascolana tenera, Tendellone and Leccino that exhibit nucleotide differences at the same position) for validation by PCR amplifications and Sanger sequencing (ABI PRISM 3130, Genetic Analyzer, Applied Biosystems™, USA) (see Supplementary Table S3). With a custom Perl script, a genomic region of 150 nucleotides surrounding each SNP *locus* was extracted and Oligo Explorer 1.2 (http://www.genelink.com/tools/gl-oe.asp) was employed to guide primer design (see Supplementary Table S3).

Linkage disequilibrium (LD) was calculated on the SNP dataset derived from the TASSEL-GBS pipeline using the SNP & Variation Suite software (SVS; v8.4.0; Golden Helix Inc. Bozeman, MT, USA, www.goldenhelix.com). LD decay across the genome was evaluated considering the SNPs of the 30 longest scaffolds (see Supplementary Table S6). The point where the loess curve reaches the plateau was considered the background level of LD.

### Genetic diversity analysis

High quality SNPs from TASSEL-UNEAK and TASSEL-GBS were used as input for a parametric [STRUCTURE v.2.3.4^67^;] and a non-parametric population structure analysis software [AWclust^68^].

As the STRUCTURE algorithm assumes independent *loci*, a SNP dataset pruned from *loci* in strong LD was generated using the SVS software v8.4.0, setting the r^2^ threshold equal to 0.5. For each K (from 1 to 10) ten independent runs were performed applying the admixture model, set a Markov chain Monte Carlo of 100,000 burn-in phases followed by 100,000 iterations. The optimal K value was estimated using Structure Harvester^94^. Cultivars having a membership coefficient (q_i_) ≥0.60 were clustered, while varieties with q_i_ < 0.60 at each assigned K were considered as admixed. To determine the level of differentiation among sub-populations, we calculated the fixation index (F_ST_) among all possible pair-wise combinations using SVS.

AWclust was also used to generate a matrix of pair-wise allele sharing distances (ADS) between all individuals in the dataset and to infer population structure. Gap statistic was employed to calculate the optimal number of groups (K) based on sample genetic relatedness^95^. Pair-wise IBS allele-sharing estimates among olive tree samples were calculated using PLINK^96^ v1.90b5.2 and graphically represented by MDS plot. TREEMIX^69^ was used to infer patterns of population mixtures and to test the presence of gene flow among olive sub-populations. A variable number of migration events (M) ranging from 0 to 10 was tested and the value of M that had the highest log-likelihood was selected as the most predictive model.

### Statistical analysis

The MSTAT-C package (1983) was used to perform a one-way analysis of variance (ANOVA) in order to determine whether there are any statistically significant differences between the means of drupe weight of cultivars in AWclust and STRUCTURE groups.

## Electronic supplementary material

Supplementary tables, figures and files

Supplementary File S1

Supplementary File S2

## Data Availability

Raw sequences were submitted to the European Nucleotide Archive (ENA; http://www.ebi.ac.uk/ena) under the study accession number PRJEB21079. Unfiltered VCF files are downloadable as compressed Supplementary Files S1 and S2.

## References

[1] Kaniewski D (2012). Primary domestication and early uses of the emblematic olive tree: palaeobotanical, historical and molecular evidence from the Middle East. Biological Reviews.

[2] Zohary, D., Hopf, M. & Weiss, E. Domestication of Plants in the Old World: The Origin and Spread of Domesticated Plants in Southwest Asia, Europe, and the Mediterranean Basin. *Oxford University Press on Demand* (2012).

[3] Besnard G., Khadari B., Navascues M., Fernandez-Mazuecos M., El Bakkali A., Arrigo N., Baali-Cherif D., Brunini-Bronzini de Caraffa V., Santoni S., Vargas P., Savolainen V. (2013). The complex history of the olive tree: from Late Quaternary diversification of Mediterranean lineages to primary domestication in the northern Levant. Proceedings of the Royal Society B: Biological Sciences.

[4] Barazani O (2016). Genetic variation of naturally growing olive trees in Israel: from abandoned groves to feral and wild?. BMC Plant Biology.

[5] Galili E, Stanley DJ, Sharvit J, Weinstein-Evron M (1997). Evidence for Earliest Olive-Oil Production in Submerged Settlements off the Carmel Coast, Israel. Journal of Archaeological Science.

[6] Besnard, G., Baradat, P., Breton, C., Khadari, B. & Berville, A. Olive domestication from structure of oleasters and cultivars using nuclear RAPDs and mitochondrial RFLPs. *Genetics Selection Evolution* **33**, S251–S268 (2001).

[7] Besnard Guillaume (2016). Origin and Domestication. Compendium of Plant Genomes.

[8] Terral JF (2004). Historical biogeography of olive domestication (*Olea europaea* L.) as revealed by geometrical morphometry applied to biological and archaeological material. Journal of Biogeography.

[9] Baldoni L (2006). Genetic Structure of Wild and Cultivated Olives in the Central Mediterranean Basin. Annals of Botany.

[10] Vossen P (2007). Olive oil: History, production, and characteristics of the world’s classic oils. HortScience.

[11] Hitchner, R. B. Olive production and the Roman economy: the case for intensive growth in the Roman Empire. *The ancient economy* 71–83 (2002).

[12] Muzzalupo I, Vendramin GG, Chiappetta A (2014). Genetic Biodiversity of Italian Olives (Olea europaea) Germplasm Analyzed by SSR Markers. The Scientific World Journal.

[13] Vivaldi GA, Strippoli G, Pascuzzi S, Stellacci AM, Camposeo S (2015). Olive genotypes cultivated in an adult high-density orchard respond differently to canopy restraining by mechanical and manual pruning. Scientia Horticulturae.

[14] Pellegrini G (2016). Application of water footprint to olive growing systems in the Apulia region: a comparative assessment. Journal of Cleaner Production.

[15] Delplancke M (2013). Evolutionary history of almond tree domestication in the Mediterranean basin. Molecular Ecology.

[16] Decroocq S (2016). New insights into the history of domesticated and wild apricots and its contribution to Plum pox virus resistance. Molecular Ecology.

[17] Cornille A (2012). New Insight into the History of Domesticated Apple: Secondary Contribution of the European Wild Apple to the Genome of Cultivated Varieties. PLOS Genetics.

[18] Diez CM (2015). Olive domestication and diversification in the Mediterranean Basin. New Phytologist.

[19] Mousavi S (2017). The eastern part of the Fertile Crescent concealed an unexpected route of olive (*Olea europaea* L.) differentiation. Annals of Botany.

[20] Myles S (2011). Genetic structure and domestication history of the grape. Proceedings of the National Academy of Sciences.

[21] Loconsole G (2016). Intercepted isolates of *Xylella fastidiosa* in Europe reveal novel genetic diversity. European Journal of Plant Pathology.

[22] Sardaro R (2016). Agro-biodiversity of Mediterranean crops: farmers’ preferences in support of a conservation programme for olive landraces. Biological Conservation.

[23] White SM, Bullock JM, Hooftman DAP, Chapman DS (2017). Modelling the spread and control of *Xylella fastidiosa* in the early stages of invasion in Apulia, Italy. Biological Invasions.

[24] Pasqualone A (2016). Evolution and perspectives of cultivar identification and traceability from tree to oil and table olives by means of DNA markers. Journal of the Science of Food and Agriculture.

[25] Belaj A, Trujillo I, De la Rosa R, Rallo L, Giménez MJ (2001). Polymorphism and discrimination capacity of randomly amplified polymorphic markers in an olive germplasm bank. Journal of the American Society for Horticultural Science.

[26] Rallo P, Dorado G, Martín A (2000). Development of simple sequence repeats (SSRs) in olive tree (*Olea europaea* L.). Theoretical and Applied Genetics.

[27] Belaj A (2007). Genetic Diversity and Population Structure of Wild Olives from the North-western Mediterranean Assessed by SSR Markers. Annals of Botany.

[28] Boucheffa S (2017). The coexistence of oleaster and traditional varieties affects genetic diversity and population structure in Algerian olive (*Olea europaea*) germplasm. Genetic Resources and Crop Evolution.

[29] Sakar E, Unver H, Ercisli S (2016). Genetic Diversity Among Historical Olive (*Olea europaea* L.) Genotypes from Southern Anatolia Based on SSR Markers. Biochemical Genetics.

[30] Alba V, Montemurro C, Sabetta W, Pasqualone A, Blanco A (2009). SSR-based identification key of cultivars of *Olea europaea* L. diffused in Southern-Italy. Scientia Horticulturae.

[31] Baldoni, L., Pellegrini, M., Mencuccini, M., Angiolillo, A. & Mulas, M. Genetic relationships among cultivated and wild olives revealed by AFLP markers. In *XXV International Horticultural Congress, Part 11: Application of Biotechnology and Molecular Biology and Breeding-Gene* **521**, 275-284, 10.17660/ActaHortic.2000.521.30 (1998).

[32] Montemurro C, Simeone R, Pasqualone A, Ferrara E, Blanco A (2005). Genetic relationships and cultivar identification among 112 olive accessions using AFLP and SSR markers. The Journal of Horticultural Science and Biotechnology.

[33] Ipek M, Seker M, Ipek A, Gul MK (2015). Identification of molecular markers associated with fruit traits in olive and assessment of olive core collection with AFLP markers and fruit traits. Genetics and Molecular Research.

[34] Kaya HB (2013). SNP discovery by illumina-based transcriptome sequencing of the olive and the genetic characterization of Turkish olive genotypes revealed by AFLP, SSR and SNP markers. PLoS One.

[35] Resta, P. *et al*. Use of AFLP to characterize Apulian olive varieties (*O. europaea* L.). *Acta Horticolturae* **586**, 73–77, 10.17660/ActaHortic.2002.586.6 (2002).

[36] Sabetta W (2013). Fad7 gene identification and fatty acids phenotypic variation in an olive collection by EcoTILLING and sequencing approaches. Plant Physiology and Biochemistry.

[37] Biton I (2015). Development of a large set of SNP markers for assessing phylogenetic relationships between the olive cultivars composing the Israeli olive germplasm collection. Molecular Breeding.

[38] Kaya HB (2016). Association Mapping in Turkish Olive Cultivars Revealed Significant Markers Related to Some Important Agronomic Traits. Biochemical Genetics.

[39] Mammadov J, Aggarwal R, Buyyarapu R, Kumpatla S (2012). SNP Markers and Their Impact on Plant Breeding. International Journal of Plant Genomics.

[40] Kim C (2016). Application of genotyping by sequencing technology to a variety of crop breeding programs. Plant Science.

[41] Torkamaneh D, Laroche J, Belzile F (2016). Genome-Wide SNP Calling from Genotyping by Sequencing (GBS) Data: A Comparison of Seven Pipelines and Two Sequencing Technologies. PLoS One.

[42] Taranto Francesca, D’Agostino Nunzio, Tripodi Pasquale (2016). An Overview of Genotyping by Sequencing in Crop Species and Its Application in Pepper. Dynamics of Mathematical Models in Biology.

[43] Verma S (2015). High-density linkage map construction and mapping of seed trait QTLs in chickpea (*Cicer arietinum* L.) using Genotyping-by-Sequencing (GBS). Scientific Reports.

[44] Boutet G (2016). SNP discovery and genetic mapping using genotyping by sequencing of whole genome genomic DNA from a pea RIL population. BMC Genomics.

[45] Heim CB, Gillman JD (2017). Genotyping-by-Sequencing-Based Investigation of the Genetic Architecture Responsible for a ∼Sevenfold Increase in Soybean Seed Stearic Acid. G3: Genes|Genomes|Genetics.

[46] Kujur A (2013). Functionally Relevant Microsatellite Markers From Chickpea Transcription Factor Genes for Efficient Genotyping Applications and Trait Association Mapping. DNA Research.

[47] Pavan Stefano, Lotti Concetta, Marcotrigiano Angelo R., Mazzeo Rosa, Bardaro Nicoletta, Bracuto Valentina, Ricciardi Francesca, Taranto Francesca, D’Agostino Nunzio, Schiavulli Adalgisa, De Giovanni Claudio, Montemurro Cinzia, Sonnante Gabriella, Ricciardi Luigi (2017). A Distinct Genetic Cluster in Cultivated Chickpea as Revealed by Genome-wide Marker Discovery and Genotyping. The Plant Genome.

[48] Taranto F, D’Agostino N, Greco B, Cardi T, Tripodi P (2016). Genome-wide SNP discovery and population structure analysis in pepper (*Capsicum annuum*) using genotyping by sequencing. BMC Genomics.

[49] Arruda Marcio P., Brown Patrick, Brown-Guedira Gina, Krill Allison M., Thurber Carrie, Merrill Keith R., Foresman Bradley J., Kolb Frederic L. (2016). Genome-Wide Association Mapping of Fusarium Head Blight Resistance in Wheat using Genotyping-by-Sequencing. The Plant Genome.

[50] Nimmakayala P (2014). Single nucleotide polymorphisms generated by genotyping by sequencing to characterize genome-wide diversity, linkage disequilibrium, and selective sweeps in cultivated watermelon. BMC Genomics.

[51] Pavan S (2017). Genotyping-by-sequencing of a melon (*Cucumis melo* L.) germplasm collection from a secondary center of diversity highlights patterns of genetic variation and genomic features of different gene pools. BMC Genomics.

[52] Catchen JM, Amores A, Hohenlohe P, Cresko W, Postlethwait JH (2011). Stacks: Building and Genotyping Loci De Novo From Short-Read Sequences. G3: Genes|Genomes|Genetics.

[53] Lu F (2013). Switchgrass genomic diversity, ploidy, and evolution: novel insights from a network-based SNP discovery protocol. PLoS Genet.

[54] Melo ATO, Bartaula R, Hale I (2016). GBS-SNP-CROP: a reference-optional pipeline for SNP discovery and plant germplasm characterization using variable length, paired-end genotyping-by-sequencing data. BMC Bioinformatics.

[55] Huang BE, Raghavan C, Mauleon R, Broman KW, Leung H (2014). Efficient Imputation of Missing Markers in Low-Coverage Genotyping-by-Sequencing Data from Multiparental Crosses. Genetics.

[56] Russell J (2014). The use of genotyping by sequencing in blackcurrant (*Ribes nigrum*): developing high-resolution linkage maps in species without reference genome sequences. Molecular Breeding.

[57] Balsalobre TWA (2017). GBS-based single dosage markers for linkage and QTL mapping allow gene mining for yield-related traits in sugarcane. BMC Genomics.

[58] Biazzi E (2017). Genome-Wide Association Mapping and Genomic Selection for Alfalfa (*Medicago sativa*) Forage Quality Traits. PLoS One.

[59] Henning JA (2016). Genotyping-by-sequencing of a bi-parental mapping population segregating for downy mildew resistance in hop (*Humulus lupulus* L.). Euphytica.

[60] İpek A (2016). SNP Discovery by GBS in Olive and the Construction of a High-Density Genetic Linkage Ma. Biochemical Genetics.

[61] Marchese A (2016). The first high-density sequence characterized SNP-based linkage map of olive (*Olea europaea* L. subsp. *europaea*) developed using genotyping by sequencing. Australian Journal of Crop Science.

[62] Sarnari, T. *ISMEA*, www.ismea.it/flex/files/7/7/d/D.d9ca1e163f12132ec6bc/Presentazione_Olio.pptx (2012).

[63] Rotondi A, Magli M, Ricciolini C, Baldoni L (2003). Morphological and molecular analyses for the characterization of a group of Italian olive cultivars. Euphytica.

[64] Hatzopoulos Polydefkis, Banilas Georgios, Giannoulia Katerina, Gazis Fotis, Nikoloudakis Nikos, Milioni Dimitra, Haralampidis Kosmas (2002). European Journal of Lipid Science and Technology.

[65] Owen CA (2005). AFLP reveals structural details of genetic diversity within cultivated olive germplasm from the Eastern Mediterranean. Theoretical and Applied Genetics.

[66] Cruz F (2016). Genome sequence of the olive tree, *Olea europaea*. GigaScience.

[67] Pritchard JK, Stephens M, Donnelly P (2000). Inference of population structure using multilocus genotype data. Genetics.

[68] Gao X, Starmer JD (2008). AWclust: point-and-click software for non-parametric population structure analysis. BMC Bioinformatics.

[69] Pickrell JK, Pritchard JK (2012). Inference of Population Splits and Mixtures from Genome-Wide Allele Frequency Data. PLOS Genetics.

[70] Godini Angelo, Vivaldi Gaetano Alessandro, Camposeo Salvatore (2011). Sidebar: Olive cultivars field-tested in super-high-density system in southern Italy. California Agriculture.

[71] Rosati A, Zipanćič M, Caporali S, Padula G (2009). Fruit weight is related to ovary weight in olive (*Olea europaea* L.). Scientia Horticulturae.

[72] Fendri M, Trujillo I, Trigui A, Rodríguez-García MI, Ramírez JDA (2010). Simple sequence repeat identification and endocarp characterization of olive tree accessions in a Tunisian germplasm collection. HortScience.

[73] Godini, A., Mariani, R., Pacifico, A. & Palasciano, M. Repeatedly reported but hitherto undescribed olive cultivars native to Southern Italy. In *IV International Symposium on Olive Growing*. **586**, 201–204, 10.17660/ActaHortic.2002.586.36 (2002).

[74] Boskou Dimitrios, Camposeo Salvatore, Clodoveo Maria Lisa (2015). Table Olives as Sources of Bioactive Compounds. Olive and Olive Oil Bioactive Constituents.

[75] Lavee S (2013). Evaluation of the need and present potential of olive breeding indicating the nature of the available genetic resources involved. Scientia Horticulturae.

[76] El Bakkali Ahmed, Haouane Hicham, Moukhli Abdelmajid, Costes Evelyne, Van Damme Patrick, Khadari Bouchaib (2013). Construction of Core Collections Suitable for Association Mapping to Optimize Use of Mediterranean Olive (Olea europaea L.) Genetic Resources. PLoS ONE.

[77] Guerin, J. & Sedgley, M. Cross-pollination in olive cultivars. *Barton: Rural Industries Research and Development Corporation* (2007)*.*

[78] Linos A, Nikoloudakis N, Katsiotis A, Hagidimitriou M (2014). Genetic structure of the Greek olive germplasm revealed by RAPD, ISSR and SSR markers. Scientia Horticulturae.

[79] Las Casas G (2014). Molecular characterization of olive (*Olea europaea* L.) Sicilian cultivars using SSR markers. Biochemical Systematics and Ecology.

[80] Terral J-F, Arnold-Simard G (1996). Beginnings of Olive Cultivation in Eastern Spain in Relation to Holocene Bioclimatic Changes. Quaternary Research.

[81] Lauri, P. E. *et al*. Does knowledge on fruit tree architecture and its implications for orchard management improve horticultural sustainability? In *I International Symposium on Horticulture in Europe* **817**, 243–250, 10.17660/ActaHortic.2009.817.25 (2008).

[82] Cherbiy-Hoffmann SU, Searles PS, Hall AJ, Rousseaux MC (2012). Influence of light environment on yield determinants and components in large olive hedgerows following mechanical pruning in the subtropics of the Southern Hemisphere. Scientia Horticulturae.

[83] Gregoriou K, Pontikis K, Vemmos S (2007). Effects of reduced irradiance on leaf morphology, photosynthetic capacity, and fruit yield in olive (*Olea europaea* L.). Photosynthetica.

[84] Albertini E (2011). Structure of genetic diversity in *Olea europaea* L. cultivars from central Italy. Molecular Breeding.

[85] Vona G (1997). The peopling of Sardinia (Italy): history and effects. International Journal of Anthropology.

[86] Malkin I (2003). Networks and the Emergence of Greek Identity. Mediterranean Historical Review.

[87] Famiani F (2014). Evaluation of different mechanical fruit harvesting systems and oil quality in very large size olive trees. 2014.

[88] Cerchiai, L., Jannelli, L. & Longo, F. The Greek Cities of Magna Graecia and Sicily. *Getty Publications* (2004).

[89] Dini A, Corretti A, Innocenti F, Rocchi S, Westerman DS (2007). Sooty sweat stains or tourmaline spots? The Argonauts on the Island of Elba (Tuscany) and the spread of Greek trading in the Mediterranean Sea. Geological Society, London, Special Publications.

[90] Khan MA, Korban SS (2012). Association mapping in forest trees and fruit crops. Journal of Experimental Botany.

[91] Doyle JJ (1990). Isolation of plant DNA from fresh tissue. Focus.

[92] Glaubitz JC (2014). TASSEL-GBS: a high capacity genotyping by sequencing analysis pipeline. PLoS One.

[93] Danecek P (2011). The variant call format and VCFtools. Bioinformatics.

[94] Earl DA (2012). & vonHoldt, B. M. STRUCTURE HARVESTER: a website and program for visualizing STRUCTURE output and implementing the Evanno method. Conservation Genetics Resources.

[95] Gao X, Martin ER (2009). Using Allele Sharing Distance for Detecting Human Population Stratification. Human Heredity.

[96] Chang CC (2015). Second-generation PLINK: rising to the challenge of larger and richer datasets. GigaScience.

