# Supplementary material for: GBS-derived SNP catalogue unveiled wide genetic variability and geographical relationships of Italian olive cultivars
Source: Sci Rep. 2018 Oct 26;8:15877. doi: 10.1038/s41598-018-34207-y (PMC6203791; doi:10.1038/s41598-018-34207-y)
Supplement: Supplementary file 1 — Supplementary tables, figures and files [file 41598_2018_34207_MOESM1_ESM.pdf]

SUPPLEMENTARY TABLES, FIGURES AND FILES FOR:

GBS-derived SNP catalogue unveiled wide genetic variability and geographical relationships of Italian olive cultivars.

Nunzio D'Agostino[1*†], Francesca Taranto[2*†], Salvatore Camposeo[3], Giacomo Mangini[4], Valentina Fanelli[2], Susanna Gadaleta[2], Monica Marilena Miazzi[4], Stefano Pavan[4], Valentina di Rienzo[2], Wilma Sabetta[2], Luca Lombardo[5], Samanta Zelasco[6], Enzo Perri[6], Concetta Lotti[7], Elena Ciani[8], Cinzia Montemurro[2,4].

[1] CREA Research Centre for Vegetable and Ornamental Crops, Pontecagnano Faiano, Italy

[2] SINAGRI S.r.l. - Spin Off of the University of Bari ''Aldo Moro'', Bari, Italy

[3] Department of Agricultural and Environmental sciences, University of Bari ''Aldo Moro'', Bari, Italy

[4] Department of Soil, Plant and Food Sciences, University of Bari ''Aldo Moro'', Bari, Italy

[5] Department of Science, University of Basilicata, Potenza, Italy

[6] CREA Research Centre for Olive, Citrus and Tree Fruit, Rende, Italy

[7] Department of the Sciences of Agriculture, Food and Environment, University of Foggia, Foggia, Italy

[8] Department of Biosciences, Biotechnologies and Biopharmaceutics, University of Bari "Aldo Moro", Bari, Italy

[*]These authors contributed equally to this work

[†]nunzio.dagostino@crea.gov.it; francesca.taranto@uniba.it

Supplementary Tables

Table S1. List of the 94 olive cultivars analysed by genotyping-by-sequencing. From left to right we reported for each cultivar possible synonyms, geographical area of cultivation, drupe weight (average weight of 100 drupes), STRUCTURE and AWclust cluster membership for SNP dataset called by TASSEL-UNEAK (T^U) and TASSEL-GBS (T^G).

Accession	Synonyms	Geographic area of cultivation	Drupe weight (g)	STRUCTURE		Awclust	
				T^U	T^G	T^U	T^G
Dolce di Cassano	/	Puglia	4.66	Adm	Adm	$I^{u}b$	I^{r}
Oliastro	Lezze, Oliva rossa; Olivastro del Gargano, Olivastro di Conversano	Puglia	2.03	Adm	Adm	$IV^{u}b$	IV^{r}
San Francesco	Sanfrancescana	Toscana/ Sicilia/ Marche/ Puglia	5.76	Adm	Adm	$IV^{u}b$	IV^{r}
Vallanella	/	Lazio	3.20	Adm	Adm	$IV^{u}b$	IV^{r}
Dritta di Loreto	Dolce, Grossa di Acquasparta, Loretana, Moscufese, San Felice	Marche	1.82	K1	K1	$I^{u}a$	II^{r}
Marzio	/	Toscana	2.25	K1	K1	$I^{u}a$	II^{r}
Pendolino	Mashio, Maurino fiorentino, Olivo passerino, Piangente	Toscana/ Marche	1.65	K1	K1	$I^{u}a$	II^{r}
Piangente	Pendolino	Toscana	2.16	K1	K1	$I^{u}a$	II^{r}
Rosciola laziale	Rosciola del Lazio	Lazio	1.71	K1	K1	$I^{u}a$	II^{r}
Americano	Firenzuolo	Toscana	2.56	K1	K1	$I^{u}a$	II^{r}
Carboncella	Roscina, Palombara, Moraiolo; Caravogna, Carbogna, Carbognola, Carbona, Carbonchio, Carbonchiola, Carboncina, Carbone, Carbonella, Marsella, Moretta, Morelo, Oliva nera, Oliva tonda, Ritornella	Lazio	1.94	K1	K1	$I^{u}a$	II^{r}
Colombina	(Leccino)	Emilia Romagna	2.39	K1	K1	$I^{u}a$	II^{r}
Leccino	Colombina, Leccio, Toscana, Verolana	Toscana	2.60	K1	K1	$I^{u}a$	II^{r}
Leccio del Corno	Grappolo	Toscana	2.30	K1	K1	$I^{u}a$	II^{r}
Maurino	Laurino, Maschio	Toscana	2.16	K1	K1	$I^{u}a$	II^{r}
Dolce di Andria	Mele	Puglia		K1	Adm	$I^{u}b$	I^{r}
Termite di Bitetto	Cima di Bitetto, Mele di Bitetto, Oliva mele, Termite	Puglia	4.16	K1	Adm	$I^{u}b$	I^{r}

Zimbimbo	Zimbimbolo	Puglia	2.12	K1	K1	$I^{u}b$	I^{r}
Frantoio	Casaliva, Correggiolo, Corsicana, Ogliarola del Bradano, Paesana, Raggia, Raggiola, Taggiasca, Razzola; Frantoiano, Gentile, Grugnola, Larcianese, Mandolina, Nostrato, Oliva lunga, Pendaglio, Radara, Raggia, Rasaro, Razzo, Resana, Sargano, Solciaro, Strangona, Vergiola	Toscana	2.38	K1	K1	$I^{u}b$	I^{r}
Ogliarola barese	Cima di Bitonto, Marinese, Ogliarola del Gargano; Baresana, Bitontina, Nostrana, Ogliarola, Paesana	Puglia	2.42	K1	Adm	$I^{u}b$	I^{r}
Taggiasca	Lavagnina, Pignola di Oneglia, Rapuina	Liguria	2.46	K1	K1	$I^{u}b$	I^{r}
Cazzinicchio	/	Puglia	3.17	K1	Adm	$IV^{u}b$	IV^{r}
Cima di Melfi	Donna Francesca	Basilicata	2.51	K1	Adm	$IV^{u}b$	IV^{r}
Coratina	Racioppa, Cima di Corato, Coratese, Olivo a grappoli, Racemo	Puglia	2.76	K1	Adm	$IV^{u}b$	IV^{r}
Cerasuola	Palermitana, Marfia; Gruppusa, Nocellara, Ogliara, Ogliarola, Olivedda, Zimmusa	Sicilia	3.25	K2	Adm	$III^{u}a$	IV^{r}
Bosana	Aliva dolci, Meliddu, Olia de Ozzu, Peranzana; Palma, Coroncina, Ogliastrina, Olieddu, Sassarese, Cerasa di Montenero	Sardegna	3.13	K2	Adm	$II^{u}a$	IV^{r}
Caiazzana	Carica, Oliva a sauce, Vernacciola	Campania	2.24	K2	K3	$II^{u}a$	$III^{r}a$
Gentile di Chieti	Paesana bianca, Rosciola di Rotello; Biancucci, Nostrana, Olivastro	Abruzzo	2.71	K2	Adm	$II^{u}a$	$III^{r}a$
Maiatica Ferrandina	Gentile, Materana, Oliva di Ferandina, Oliva dolce, Paesana, Pasola	Basilicata	3.70	K2	K3	$II^{u}a$	$III^{r}a$
Nebbio di Chieti	Comune, Gentile, Ordinaria	Abruzzo	2.36	K2	Adm	$II^{u}a$	$III^{r}a$
Ogliarola del Vulture	/	Basilicata	1.83	K2	K1	$II^{u}a$	$III^{r}a$
Ortolana	Bella di san Lorenzo maggiore, Melella	Campania	4.31	K2	Adm	$II^{u}a$	$III^{r}a$
Pampagliosa	/	Campania	3.33	K2	Adm	$II^{u}a$	$III^{r}a$
Pignola	Pinola, Arbequina mendocina, Crova, Liguria, Merletta, Negretta, Olivo del povero, Pigneux, Pignola, Premice	Liguria	2.41	K2	Adm	$II^{u}a$	$III^{r}a$
Pisciottana	Ogliastrina, Olivo dell'ascea,	Campania	2.05	K2	Adm	$II^{u}a$	IV^{r}
Puntella	/	Abruzzo	2.06	K2	Adm	$II^{u}a$	$III^{r}a$

Racioppella	Racioppa, Bella di san Lorenzo, Cacaccella, Favecotte, Grappetella, La ricca, Olivo a raciuppe, Racioppella di guardia Sanframondi, Rappocella, Spruarella	Campania	2.49	K2	Adm	$II^{u}a$	$III^{r}a$
Raja	Oliva dolce, olivo di Macciano, Ortana, Raggia, Razza, Secolare di Giano	Umbria/ Lazio/ Marche	2.32	K2	Adm	$II^{u}a$	$III^{r}a$
Rosciola	Caprigna, ogliarola, Ragiola, Risciola, Rossa, Rossaia, Rossolo, Rusciola, Rosciola del Lazio	Lazio/ Marche/ Toscana	1.74	K2	Adm	$II^{u}a$	$III^{r}a$
Tendellone	Fecciaro, Pallottona, Raggio bastardo, Fezzalupo (Catanzaro)	Umbria	3.77	K2	Adm	$II^{u}a$	IV^{r}
Zinzifarica	/	Calabria	3.50	K2	K3	$II^{u}a$	$III^{r}a$
Bottoni di Gallo	Sperone di gallo, Oliva torta	Molise	1.46	K2	K2	$II^{u}b$	$III^{r}b$
Cima di Mola	Ogliarola di Lecce, Chiarita, Cima di Fasano, Cima di Mola, Cima di Monopoli, Fasanese, Giuggiolara, Mennedda, Molese, Monopolese, Nasuta, Ogliarola salentina, Pizzuta, Salentina, Tarantina	Puglia	1.43	K2	K2	$II^{u}b$	$III^{r}b$
Mignola	Laurina, Sargana, Sarganella, Suricina; Carbonella, Laura, Migliola, Mignolo, Mindola, Mindulina, Miola, Sargano	Marche	1.53	K2	K2	$II^{u}b$	$III^{r}b$
Canino	Caninese, Oliva di Canino, Montignoso	Lazio	1.29	K2	K2	$II^{u}b$	$III^{r}b$
Cellina di Nardò	Asciulo, Cafredda, Gasciola, Inchiastra, Leccese, Morella, Oliva di Lecce, Oliva di Nardò, Saracena, Scorrano, Vosciola	Puglia (Libia)	1.39	K2	K2	$II^{u}b$	$III^{r}b$
Ciciarello	Ciccarella, Olivo amaro, Olivo minuto, Resta	Calabria	1.87	K2	K2	$II^{u}b$	$III^{r}b$
Dolce Agogia	Nerello, Olivella	Umbria	2.19	K2	Adm	$II^{u}b$	$III^{r}a$
Ottobratica	Probably is an libyan origin (Gharyan)/ Dolce, Mirtoleo, Perciatella	Calabria	1.67	K2	K2	$II^{u}b$	$III^{r}b$
Rastellina	Rastrellina	Umbria/ Toscana	1.67	K2	K2	$II^{u}b$	$III^{r}b$
Sargano	Frantoio	Toscana	1.49	K2	K2	$II^{u}b$	$III^{r}b$
Sinopolese	Calabrese, Chianota, Coccitano, Oleastrela, Pizzocorno, Scialorea, Seminara	Calabria	1.66	K2	K2	$II^{u}b$	$III^{r}b$
Pasola	Maiatica, Fasola, Pasola d'Andria	Puglia/ Basilicata	3.73	K2	Adm	$I^{u}b$	I^{r}
Toscanina	Oliva a grappa	Puglia	1.64	K2	Adm	$I^{u}b$	I^{r}
Bianchella	Bianchetta, Bianchella del Trasimeno	Umbria	2.32	K2	Adm	$I^{u}b$	I^{r}

Salvia	/	Lazio	1.66	K2	Adm	$I^{u}b$	I^{r}
Frangivento	Cipressino, Olivo di Pietrafitta	Puglia	2.71	K2	Adm	$IV^{u}b$	IV^{r}
Marina	Ogliarola barese	Lazio	2.71	K2	Adm	$IV^{u}b$	IV^{r}
Monaca	Calabrese, Monachella	Sicilia	4.27	K2	Adm	$IV^{u}b$	IV^{r}
Nociara	Fra' Michele	Puglia	2.70	K2	Adm	$IV^{u}b$	IV^{r}
Racioppa	Spinoso, Cioppella del Melfese, di Venosa, Racioppa di Rivello, di Vietri, Racioppella di Lavello, di Venosa	Basilicata	3.50	K2	Adm	$IV^{u}b$	IV^{r}
Simona	Simone	Puglia	1.71	K2	K2	$IV^{u}b$	IV^{r}
Ascolana Tenera	Noce, Oliva dolce, Oliva grossa, Oliva noce, Spanish Queen	Marche	7.34	K3	Adm	$III^{u}a$	V^{r}
Borgese	Carolea	Calabria	8.65	K3	Adm	$III^{u}a$	V^{r}
Carolea	Catanzarese, Becco di corvo, Borgese, Colarè, Convitè, Coriolese, Cortalese, Cumignana, Nicastrese, Oliva dolce, Olivona, Policastrese, Squillaciota, Verdella	Calabria	5.96	K3	Adm	$III^{u}a$	V^{r}
Giarraffa	Nocellara del Belice, Ogliarola messinese, Tonda iblea; Becco di corvo, Cefalutana, Giardara, Giuliana, Mammona, Pizzo di corvo	Sicilia	6.67	K3	K4	$III^{u}a$	V^{r}
Grappolo	Leccio del corno	Toscana	2.53	K3	Adm	$III^{u}a$	IV^{r}
Moresca	Jannusa, Minnulara, Morghetana, Nerba; Bianculidda, Catanisa, Francofontese, Giandalora, Imperiale, Maioana, Marsalisa, Mendolara, Nostrale, Ogliarola, Passulunara, Polina, Reale, Tortella, Ugghialara	Sicilia	4.58	K3	Adm	$III^{u}a$	V^{r}
Nocellara etnea	Bianca, Forte, Ghiandolara, Marmorina, Nerba catanese, Patornese, Pizzuta, Rappara, Tortella, Verdesca, Verdese	Sicilia	4.81	K3	Adm	$III^{u}a$	V^{r}
Ogliarola messinese	Castriciana, Calamigna, Castranese, Giarraffa, Messinese, Nostrale, Oliva di Termini, Passuliuara, Pattese, Paturnisa, Pllinese, Raffa, Terminese	Sicilia	5.87	K3	Adm	$III^{u}a$	V^{r}
Orbetana	Marchigiana; Corva, Oliva di san Franceso, Razzo, Sarga, Urbitana	Marche	4.51	K3	Adm	$III^{u}a$	V^{r}
Pennullara	Nostrale di Caccuri	Calabria		K3	Adm	$III^{u}a$	V^{r}

Piantone Mogliano	Limoncella, Oliva riccia, Piantone di Macerata, Raggiola, Rosciola, Rosciola di Tolentino	Marche	3.05	K3	Adm	$III^{u}a$	V^{r}
Pizz'e Carroga	Becco di Cornacchia, Bianca, Olia bianca, Oliva di Villacidro, Puntuda, Sarda	Sardegna		K3	Adm	$III^{u}a$	V^{r}
Romanella	Rotondella	Calabria	4.93	K3	Adm	$III^{u}a$	V^{r}
Sant'Agostino	Oliva grossa, Grossa di Andria, Oliva di Andria, Oliva pane, Cazzarola	Puglia	3.61	K3	Adm	$III^{u}a$	V^{r}
Tonda di Filadelfia	/	Calabria	3.60	K3	Adm	$III^{u}a$	V^{r}
Tonda Iblea	Marmorina; Caloria, Cetral, Ferlese, Comune, Giarraffa; Grossaia, Nuciddara, Prunara, Raffiuna, Siracusna, Tunna, Vitriolo	Sicilia	5.49	K3	Adm	$III^{u}a$	V^{r}
Nera di Gonnos	Maiorca (1078/ 1363), Confetto, Tonda di Cagliari; Niedda, Oliva di Gonnos	Sardegna	6.30	K3	Adm	$III^{u}b$	V^{r}
Nocellare messinese	Nuciddara, Turdunazza antimosca, Verdella	Sicilia	5.68	K3	Adm	$III^{u}b$	V^{r}
Tonda di Cagliari	Majorca, Nera di Gonnos; Tonda Coma de Ilisci	Sardegna	5.84	K3	Adm	$III^{u}b$	V^{r}
Semidana	Terza, Olia de Riola, Olivo terzo grande	Sardegna	4.43	K3	Adm	$II^{u}a$	IV^{r}
Cerasella	Mele, Amele, Annolca, Culmona, Dolce, Dolce di Andia, Dolce di Puglia, Dolce di Sannicandro, Dolce Mele, Dolce piccola, Innolache, Noce, Nolca, Nura, Oliva nocella, Oliva roccella	Puglia	3.86	K3	Adm	$IV^{u}a$	I^{r}
Mele	Cerasella, Dolce di Andria, Nolca, Dolce	Puglia		K3	Adm	$IV^{u}a$	I^{r}
Mora	/	Puglia	5.01	K3	Adm	$IV^{u}a$	I^{r}
Nolca	Mele	Puglia	3.55	K3	Adm	$IV^{u}a$	I^{r}
Augellina	Angellina, Cellina delle Murge, Cellina di Cassano	Basilicata	4.57	K3	Adm	$IV^{u}b$	IV^{r}
Calatina	Galatina, Montagna	Sicilia	3.48	K3	Adm	$IV^{u}b$	IV^{r}
Cassanese	Grossa di Cassano, Precoce di Cassano	Calabria	3.93	K3	Adm	$IV^{u}b$	IV^{r}
Corniola	Cornale, Cornulara, Farisana, Oliva minuta, Passula	Calabria/ Puglia/ Croazia	3.26	K3	Adm	$IV^{u}b$	IV^{r}
Crastu	Cristoraffo, Pizzulidda, Rizziteddu	Sicilia		K3	Adm	$IV^{u}b$	IV^{r}

Itrana	Aitana, Cicerone, Gaetana, Esperia, Oliva di Gaeta, Oliva di Itri, Oliva di Formia, Oliva grossa, Strano, Tanella, Tanesca, Velletrana	Lazio	3.78	K3	Adm	$IV^{u}b$	IV^{r}
San Benedetto	Sanbenedettese Termitana	Sicilia	3.84	K3	Adm	$IV^{u}b$	IV^{r}
Tondina	Ottobratica rotondella, Roggianella, Cicinella, Amaro, Gaetana, Marinella, Oliva di Cariati, di Cosmo albanese, Spezzanota, Vernile	Calabria	2.88	K3	Adm	$IV^{u}b$	IV^{r}

Table S2. Mean squares of olive drupe weight of cultivars in AWclust and STRUCTURE groups derived from two different SNP calling pipeline (TASSEL-UNEAK and TASSEL-GBS)

Source of variation	d.f.	TASSEL-UNEAK		d.f.	TASSEL-GBS	
		STRUCTURE	AWclust		STRUCTURE	AWclust
Between clusters	3	37.922***	34.173***	4	17.726***	29.156***
Within cluster	85	1.007	1.139	84	1.529	0.985

*** Significant differences at 0.001 P value; d.f.= degrees of freedom

Table S3. List of ten SNP *loci* selected for validation by PCR amplifications and Sanger sequencing in three cultivars.

Marker	Alleles	Scaffold	Position	Forward Primer 5'-3'	Reverse primer 5'-3'	T_m	Product size (bp)	Ascolana Tenera	Leccino	Tendellone
S1_5476481	A/T	OE6_S00005	420334	AAGAAAAGCAAAATGCCTCG	CATTTCGTGTCATTGCTCGT	53	164	n.a.	T	A
S1_5746249	C/T	OE6_S00005	690102	TTGGAGACTTTTATGCTCAC	TAGGTAAGTGGGTTCAGTAA	54	205	Y	C	T
S1_6673237	C/G	OE6_S00006	112909	TGCACCAATGTAGCCATCAA	TTTCATCAGGTTTTTTCACG	53	205	C	G	S
S1_9032471	C/A	OE6_S00007	1010134	GAGCCAAGAACACAAGTGCA	AGACGTTTTGTTCCCTGTGC	59	233	M	A	C
S1_11378373	C/A	OE6_S00009	638848	AAATACCACGTGTGCCTGTG	CAAGTGCGATTCTATGGGGC	58	163	C	A	M
S1_1112173174	G/A	OE6_S09619	463721	TCTCTCGAGCAATGCCAAAC	TGTTGTGGCCTATTTTATGCATT	57	212	A	R	G
S1_1160752348	A/G	OE6_S09678	373891	GGTGAATCCCATAAAATGTGCG	TGGGGTAACTTCAGGTCGTC	58	221	G	R	A
S1_1225811364	A/T	OE6_S09824	82650	TGTGGTCGAAAAGGAATTGCA	TGGCTTTGTGGGTCCTATTCT	58	196	T	W	n.a.
S1_1232287062	C/G	OE6_S09844	260124	TCGCAAATTGTTAATGCACGA	GAGGTCAATCTTCAAAAGCATCG	58	202	C	G	S
S1_1235619693	A/T	OE6_S09855	137493	TGGAGGGGTTGTTTAAGAAGGA	ACTCTTCCTTGCCGAATATGC	58	177	A	A	T

n.a.= not available

Table S4. List of pairs of cultivars with identity-by-state (IBS) allele-sharing estimates >0.95. Stars indicate novel cases of synonymy.

Cultivar A	Cultivation Area	Cultivar B	Cultivation Area	IBS
Borgese	Calabria	Pennullara	Calabria	0.980
Bottoni di Gallo*	Molise	Cima di Mola	Apulia	0.951
Bottoni di Gallo*	Molise	Mignola	Marche	0.966
Cerasella	Apulia	Mele	Apulia	0.957
Cerasella	Apulia	Nolca	Apulia	0.953
Cima di Mola*	Apulia	Mignola	Marche	0.962
Dolce di Andria	Apulia	Termine di Bitetto	Apulia	0.954
Dritta di Loreto*	Marche	Marzio	Tuscany	0.981
Dritta di Loreto*	Marche	Rosciola laziale	Lazio	0.982
Frantoio	Tuscany	Ogliarola barese	Apulia	0.977
Frantoio	Tuscany	Taggiasca	Liguria	0.983
Marzio*	Tuscany	Rosciola laziale	Lazio	0.980
Mele	Apulia	Nolca	Apulia	0.963
Nebbio di Chieti*	Abruzzo	Pignola	Liguria	0.971
Nera di Gonnos	Sardinia	Nocellare messinese	Sicily	0.980
Nera di Gonnos	Sardinia	Tonda di Cagliari	Sardinia	0.977
Nocellare messinese	Sicily	Tonda di Cagliari	Sardinia	0.981
Ogliarola barese	Apulia	Taggiasca	Liguria	0.976
Pendolino*	Tuscany	Piangente	Tuscany	0.975

Table S5. Analysis of the TREEMIX log-likelihood values for 0 to 3 migrations revealed that b the highest log-likelihood 2 migration events were assumed. Relative migration weights are indicated.

**M**	**ln(lik)**	**Added migration events**	
0	-892,18498		
1	-30,414103	0.269 C3^u → C4^u	
2	80,178093	0.278 C3^u → C4^u	0.448 C1^u → C4^u
3	80,207574	0.277 C3^u → C4^u	0.487 C1^u → C4^u

Table S6. List of the 30 longest olive scaffolds including SNPs used for LD decay estimation.

**Scaffold ID**	**Lenght (bp)**
Oe6_s01922	2580346
Oe6_s03843	2536859
Oe6_s05764	2523920
Oe6_s07686	2240446
Oe6_s09607	2096410
Oe6_s09608	2089551
Oe6_s07687	1923341
Oe6_s05765	1885776
Oe6_s03844	1855288
Oe6_s01923	1842151
Oe6_s00002	1827728
Oe6_s07688	1794324
Oe6_s09609	1710228
Oe6_s05766	1709168
Oe6_s03845	1695730
Oe6_s01924	1676923
Oe6_s00003	1664807
Oe6_s09610	1647348
Oe6_s07689	1631851
Oe6_s03846	1601848
Oe6_s09611	1570811
Oe6_s00004	1563312
Oe6_s07690	1555990
Oe6_s05768	1547260
Oe6_s03847	1528064
Oe6_s01926	1506713
Oe6_s00005	1504081
Oe6_s09612	1487753
Oe6_s07691	1478965
Oe6_s03848	1469283

Table S7. Potential cases of synonymy identified by allele frequency estimates on 94 Italian olive varieties. Stars indicate novel cases of synonymy.

**Possible cases of synonymy**	**Cultivation area**
Cerasella, Mele and Nolca	Apulia
Rosciola Laziale and Marzio*	border between Lazio and Tuscany
Pennulara and Borgese	Calabria
Maiatica di Ferrandina and Zinzifarica*	Basilicata and Calabria
Ogliarola Messinese, Nocellara Messinese and Sant'Agostino*	Sicily and Apulia
Nera di Gonnos, Tonda di Cagliari and Tonda Iblea	Sardinia and Sicily
Cima di Mola, Bottoni di Gallo and Mignola*	Apulia, Molise and Marche
Ogliarola barese, Taggiasca and Frantoio	Apulia, Liguria and Tuscany

Supplementary Figures

Figure S1. Bar chart describing the distribution across 94 olive cultivars of SNP types (%) called by TASSEL-UNEAK and TASSEL-GBS.

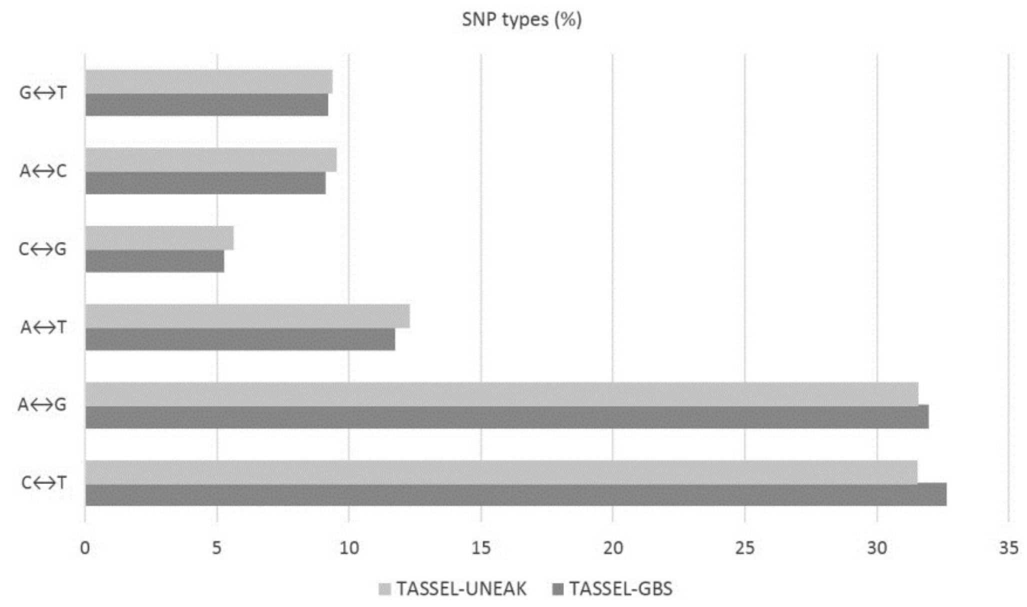

Figure S2. Stacked bar charts describing the distribution of 8,088 and 37,792 high quality SNPs called by TASSEL-UNEAK (A) and TASSEL-GBS (B) in the following categories: reference allele, alternate allele, heterozygous allele and missing values.

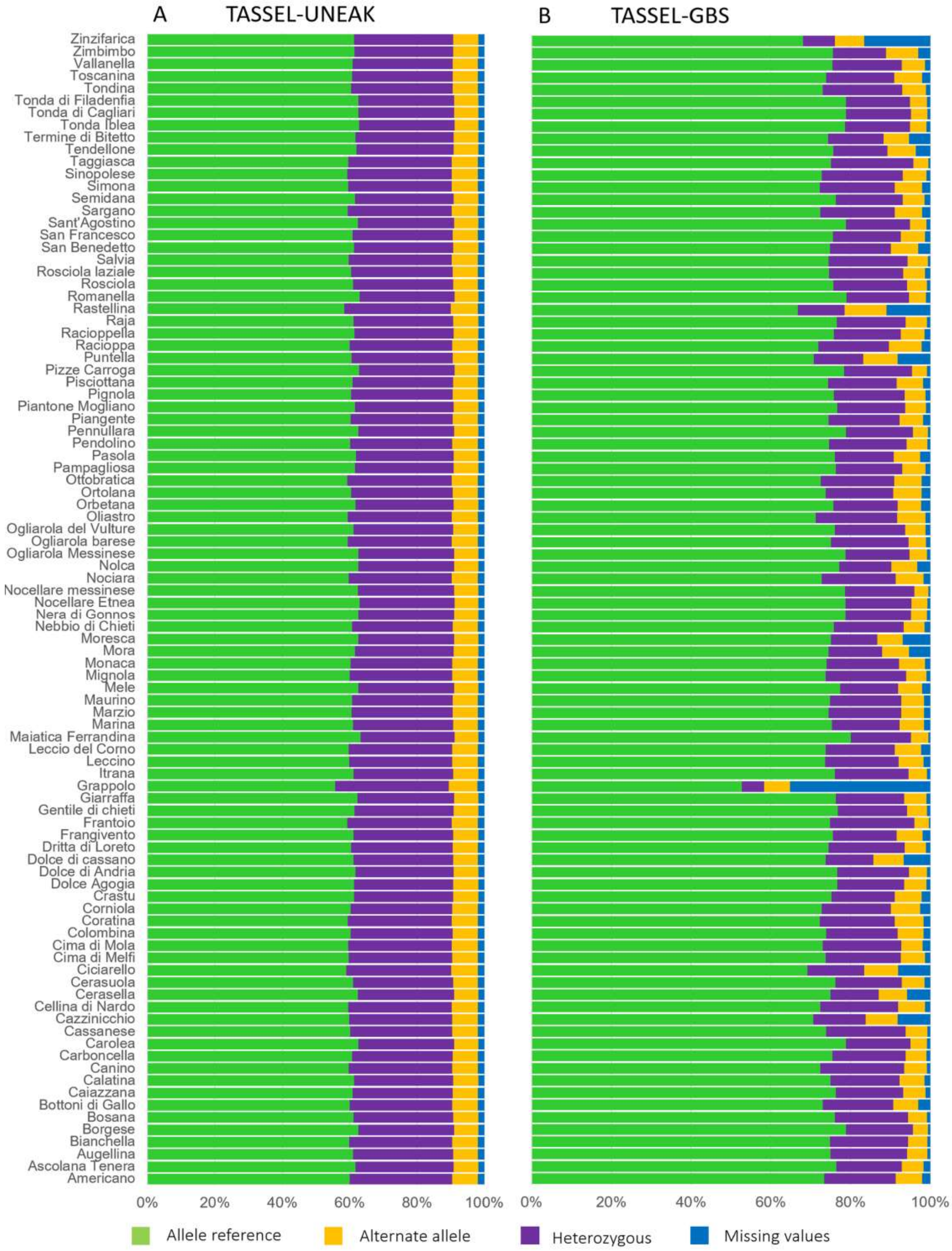

Figure S3. A) Evaluation of the best number of genetic clusters (K) using Evanno's method for population structure inferred from SNPs called by TASSEL-UNEAK. On the left it is shown the plot of mean likelihood L(K) and variance for 10 independent runs for each value of K (2-10); on the right it is reported Evanno's plot generated by STRUCTURE HARVESTER for the detection of the most likely value of K. The best value was at K=3, portioning the 94 olive cultivars into 3 genetic clusters. B) Evaluation of the best grouping number (K) using Evanno's method for population structure inferred from SNPs called by TASSEL-GBS. On the left it is shown the plot of mean likelihood L(K) and variance for 10 independent runs for each value of K (1-15); on the right it is reported Evanno's plot generated by STRUCTURE HARVESTER for the detection of the most likely value of K. The highest value was at K=4, indicating that the 94 cultivars likely form 4 sub-populations.

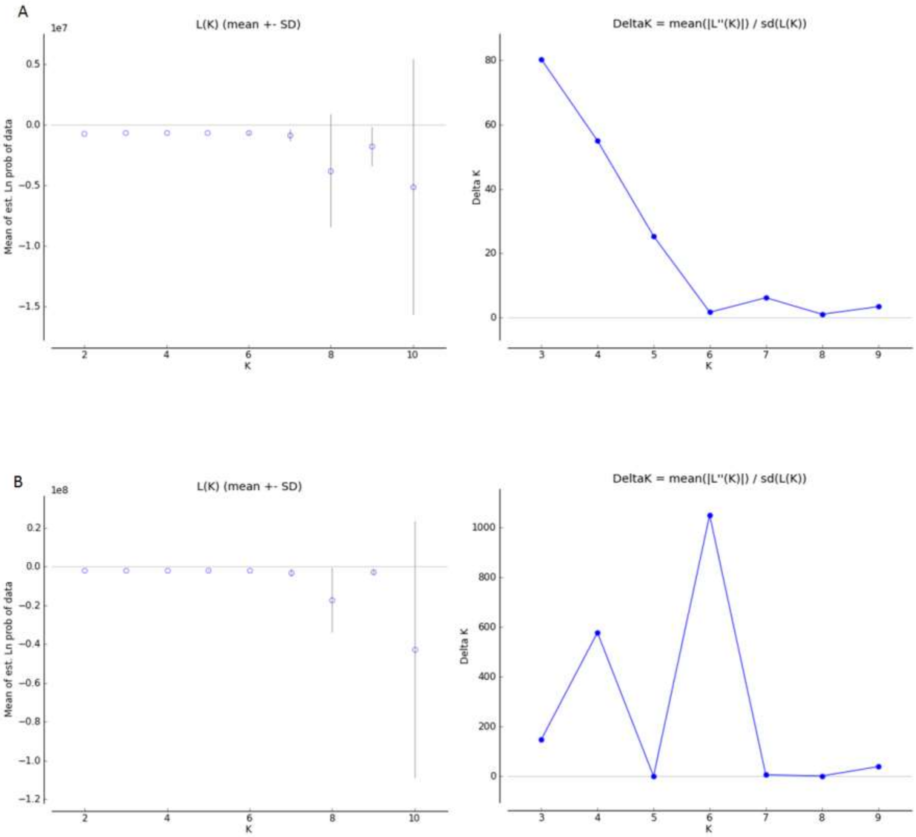

Figure S4. Gap statistic plots to estimate the best number of genetic cluster (K) in two populations whose structure was derived using SNP markers called by TASSEL-UNEAK (A) and TASSEL-GBS (B). The number of inferred Ks ranging from 1 to 15 is shown in the graphs. The estimated expectation of log (Wk) and the observed log (Wk) are indicated by the blue and red curves, respectively. As shown by the x- and y-axes representing different possible Ks and the corresponding gap values, individuals in both populations could be partitioned into K=4 (A) and K=5 (B) genetic clusters.

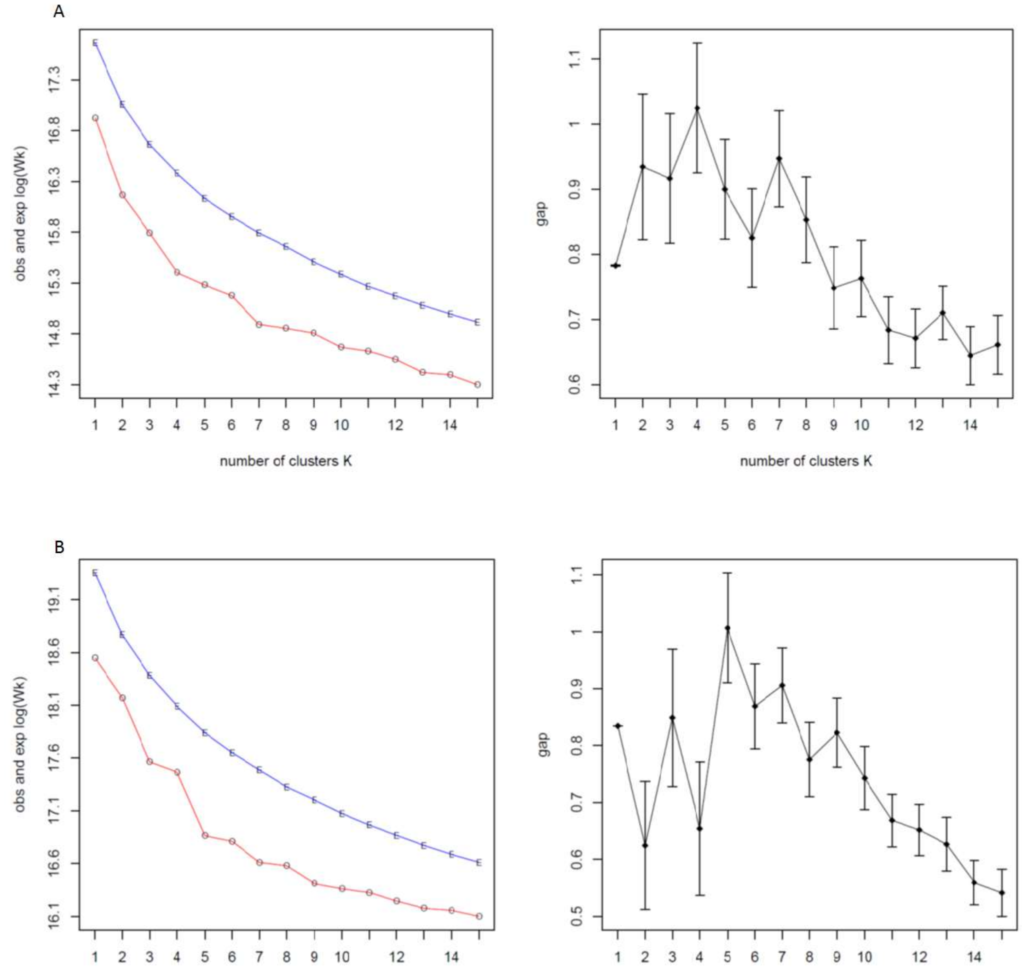

Figure S5. Multi-dimensional scaling (MDS) plot of the IBS distance matrix calculated using 22,088 unlinked SNPs (TASSEL-GBS SNP calling pipeline). Colours refer to different sub-populations. The 94 olive cultivars were clearly grouped into three clusters, while members of two other groups (i.e. Ir and IVr) are scattered in the multidimensional space. The clustering pattern is collinear with the population structure as inferred by STRUCTURE and AWclust.

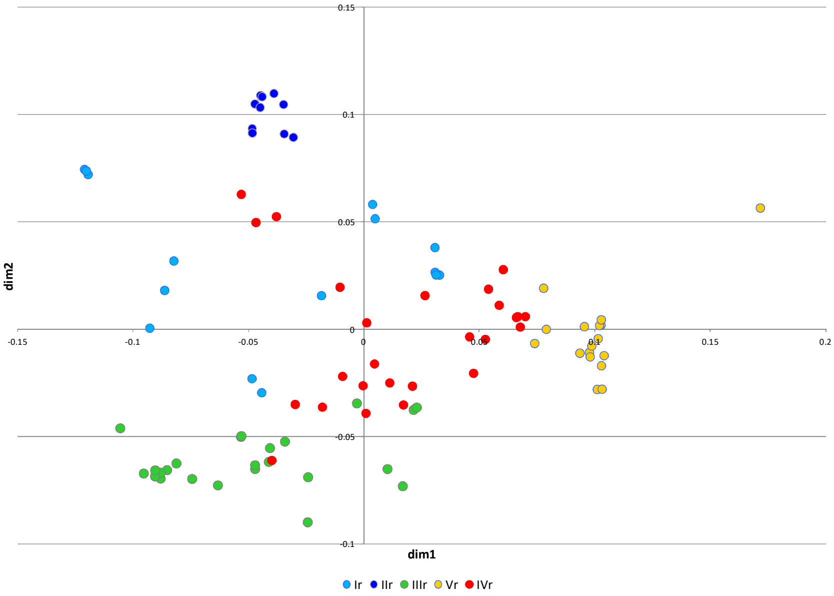

Supplementary Files

File S1. Unfiltered VCF file including SNPs called by the TASSEL-GBS pipeline.

File S2. Unfiltered VCF file including SNPs called by the TASSEL-UNEAK pipeline.
